# ANGPTL4 Exacerbates Renal Injury in Diabetic Kidney Disease by Impairing Podocyte Lipophagy via Compromised Lysosomal Degradative Function

**DOI:** 10.1002/advs.76771

**Published:** 2026-07-27

**Authors:** Xiaojing Liu, Shimin Jiang, Zhenkun Yang, Shunlai Shang, Jiayi Li, Guming Zou, Cheng Zhou, Wenge Li

**Affiliations:** ^1^ Department of Nephrology China‐Japan Friendship Hospital Beijing China; ^2^ China‐Japan Friendship Hospital (Institute of Clinical Medical Sciences) Chinese Academy of Medical Sciences and Peking Union Medical College Beijing China; ^3^ Health Science Center Peking University Beijing China

**Keywords:** ANGPTL4, diabetic kidney disease, lipophagy, lysosomal dysfunction, podocytes

## Abstract

Diabetic kidney disease (DKD) progression is closely linked to the loss of podocyte homeostasis, driven in part by intracellular lipid accumulation and impaired autophagic clearance. This study identifies angiopoietin‐like protein 4 (ANGPTL4) as a potential regulator of podocyte lipophagy in DKD. In renal biopsies from patients with DKD, ANGPTL4 is upregulated in podocytes, and its expression is associated with greater proteinuria and more rapid renal function decline. In immortalized human podocytes, exposure to high glucose and palmitic acid (HGPA) increases both intracellular and secreted ANGPTL4, impairs autophagic flux, and promotes lipid droplet accumulation. Mechanistically, ANGPTL4 overexpression reduces TFEB nuclear localization, increases lysosomal pH, and decreases cathepsin B and lysosomal acid lipase activities, consistent with impaired terminal lysosomal lipid degradation. Conversely, ANGPTL4 knockdown restores autophagic and lysosomal programs under HGPA conditions. Recombinant human N‐terminal ANGPTL4 fragment reproduces several TFEB‐localization, lysosomal, and lipophagic defects, whereas extracellular neutralization partially reverses these changes. Expression of constitutively active TFEB‐S211A restores lysosomal function and lipid clearance and attenuates profibrotic remodeling. Collectively, these findings support an ANGPTL4–TFEB‐associated lysosome–lipophagy pathway linking diabetic metabolic stress to defective lipid clearance and podocyte injury.

## Graphical abstract

In diabetic kidney disease, elevated podocyte ANGPTL4 is linked to reduced TFEB nuclear localization and compromised lysosomal degradative function. These changes impair podocyte lipophagy and promote lipid‐droplet accumulation and podocyte injury, which may contribute to renal injury progression. Extracellular N‐terminal ANGPTL4 recapitulates key abnormalities, whereas ANGPTL4 neutralization partially attenuates these effects and TFEB‐S211A produces a more pronounced rescue.

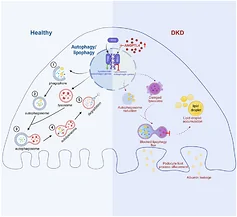

## 1 Introduction

Diabetic kidney disease (DKD) is one of the most common microvascular complications of diabetes mellitus and remains a leading cause of end‐stage kidney disease worldwide [1, 2]. Despite advances in standard‐of‐care management and the achievement of recommended glycemic and blood pressure targets, substantial residual risk of DKD progression persists among patients with type 2 diabetes [3, 4, 5, 6, 7]. This sustained clinical progression is closely linked to the irreversible injury and depletion of glomerular podocytes—highly specialized epithelial cells with intrinsically limited regenerative capacity [8, 9, 10]. Podocyte depletion is a well‐established pathological hallmark and a key driver of chronic kidney disease progression in both clinical and experimental settings [11, 12, 13, 14]. Therefore, elucidating the molecular mechanisms that disrupt podocyte homeostasis under diabetic conditions is essential for developing early interventions to prevent irreversible glomerular injury.

Maintaining podocyte homeostasis depends on efficient intracellular clearance systems, including autophagy and lysosomal degradation, to manage metabolic stress [15, 16, 17, 18]. Disruption of these processes, particularly in the context of diabetic lipotoxicity, promotes intracellular lipid accumulation and cellular dysfunction, thereby contributing to podocyte injury and glomerular damage. Lipophagy, the selective autophagic degradation of lipid droplets, is an important component of intracellular lipid homeostasis, yet its regulation in diabetic podocytes remains poorly understood [17, 19, 20]. Among the factors implicated in metabolic dysregulation, angiopoietin‐like protein 4 (ANGPTL4) has attracted considerable attention. ANGPTL4 is a secreted glycoprotein that regulates systemic lipid metabolism through inhibition of lipoprotein lipase, and circulating ANGPTL4 levels are associated with cardiovascular disease, type 2 diabetes, and obesity [21, 22, 23, 24, 25]. Within the kidney, however, ANGPTL4 exhibits distinct cell type‐ and disease‐specific functions [26, 27, 28, 29]. Pioneering work identified podocyte‐derived, poorly sialylated ANGPTL4 as a key mediator of severe proteinuria and foot process effacement in minimal change disease [26, 27]. Furthermore, renal Angptl4 expression is significantly increased in diabetic mouse models, and genetic ablation of *Angptl4* attenuates diabetes‐associated kidney fibrosis, strongly implicating it in DKD progression [14, 30, 31, 32].

However, evidence defining the expression pattern and functional relevance of ANGPTL4 in human DKD kidney biopsies remains limited. It is also unclear whether podocyte injury is mediated by intracellular ANGPTL4, extracellular secreted ANGPTL4, or both. Most importantly, the pathway linking ANGPTL4 to lysosomal dysfunction and defective lipid clearance remains incompletely defined. To address these questions, we first validated ANGPTL4 upregulation in podocytes from human DKD biopsies and examined its association with disease severity indices. Using in vitro models of diabetic lipotoxicity, we then showed that ANGPTL4 overexpression impairs autophagic flux and promotes lipid droplet accumulation, whereas ANGPTL4 knockdown restores autophagic and lysosomal programs. Mechanistically, ANGPTL4 increases transcription factor EB (TFEB) Ser211 phosphorylation, reduces the nuclear localization of TFEB, increases lysosomal pH, and decreases cathepsin B and lysosomal acid lipase activities, thereby compromising terminal lysosomal lipid degradation. Recombinant human N‐terminal ANGPTL4 fragment reproduces several lysosomal and lipophagic abnormalities, whereas extracellular neutralization partially reverses them. Moreover, constitutively active TFEB‐S211A restores lysosomal function and lipid clearance and attenuates podocyte profibrotic remodeling. Collectively, our findings support a functional ANGPTL4‐TFEB‐associated lysosome‐lipophagy pathway linking diabetic metabolic stress to defective lipid clearance and podocyte injury.

## 2 Results

### 2.1 Podocyte ANGPTL4 Upregulation Is Associated With Proteinuria and Disease‐Severity Indices in Diabetic Kidney Disease

To determine ANGPTL4 expression in human DKD, we screened 553 confirmed cases over 15 years. After excluding patients with non‑DKD glomerulopathies or insufficient follow‑up (<1 year), we randomly selected a final cohort of 30 patients (Figure S1; baseline characteristics in Table 1). Immunohistochemical staining of kidney biopsies revealed widespread ANGPTL4 expression in DKD tissues, with strong signals in glomeruli and vascular endothelial cells and weaker expression in the tubulointerstitium (Figure 1A). Co‑staining with the podocyte marker nephrin confirmed ANGPTL4 expression within podocytes (Figure 1B). Quantitative analysis showed partial colocalization (Pearson's coefficient: 0.65 in DKD vs. 0.53 in controls, not significant), which persisted despite reduced nephrin signal in DKD (Figure 1C). Semi‑quantitative analysis demonstrated a significant increase in ANGPTL4 levels in DKD glomeruli (∼3‑fold) and tubulointerstitium (∼4‑fold) compared to controls (Figure 1D,E).

**TABLE 1:** Clinical Characteristics of DKD Patients.

Clinical Characteristics	
Demographic Data	n = 30
Male, n/N (%)	21/30 (70.0)
Age (years)	49.37 ± 10.37
Medical History	
Type 2 diabetes, n/N (%)	30/30 (100.0)
Duration of diabetes (months)	121.41 ± 65.31
Diabetic retinopathy, n/N (%)	23/30 (76.67)
Hypertension, n/N (%)	27/30 (90.0)
Systolic BP at biopsy (mmHg)	132.32 ± 12.19
Nephrotic syndrome, n/N (%)	8/30 (26.67)
Cardiovascular disease, n/N (%)	8/30 (26.67)
Laboratory Tests	
WBC count (×10^9^/L)	6.89 ± 3.63
Hemoglobin (g/L)	109.02 ± 19.76
Serum creatinine at biopsy (µmol/L)	140.15 ± 55.91
eGFR (mL/min/1.73 m^2^)	54.36 ± 23.51
Serum albumin (g/L)	37.12 ± 6.39
24‐h UTP (g/24 h)	2.25 (1.48 – 4.55)
Follow‐up	
Follow‐up duration (months)	21.5 (12.00 – 31.25)

Abbreviations: BP, blood pressure; eGFR, estimated glomerular filtration rate; UTP, urinary total protein; WBC, white blood cell

**FIGURE 1:**
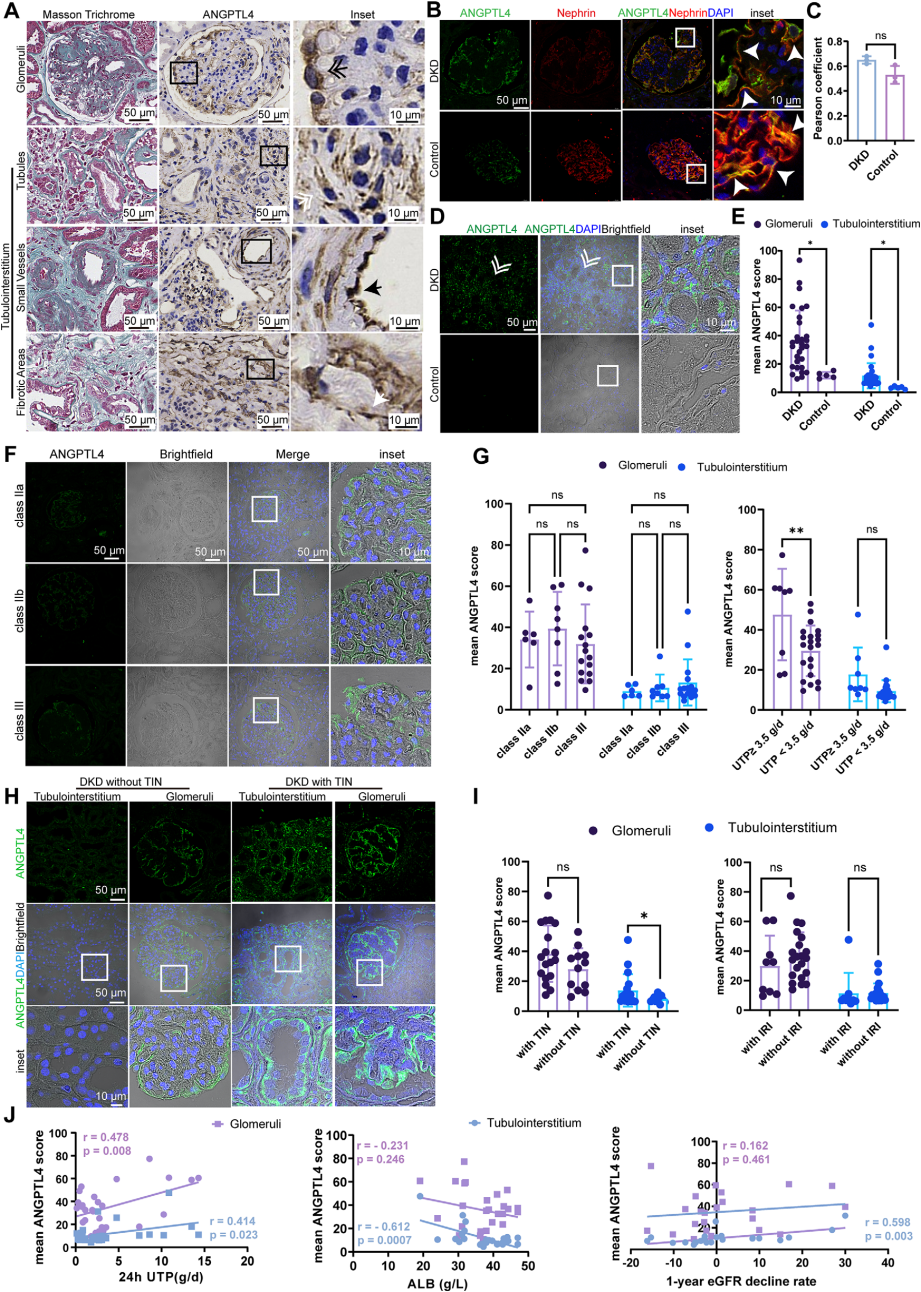
ANGPTL4 expression is upregulated and associated with disease‐severity indices in human diabetic kidney disease. (A) Representative images of Masson's trichrome staining and immunohistochemical staining for ANGPTL4 in renal tissues from DKD patients. From top to bottom: ANGPTL4 expression in glomeruli, renal tubules, interstitial microvessels, and fibrotic areas. Black double arrows: podocytes; white double arrows: tubular cells; black arrows: microvascular endothelial cells; white arrows: fibroblasts. Scale bar = 50 µm; insets = 10 µm. (B, C) Expression and colocalization of ANGPTL4 with the podocyte marker nephrin. (B) Immunofluorescence co‑staining of ANGPTL4 (green) and nephrin (red) in glomeruli; nuclei are counterstained with DAPI (blue). White arrows indicate sites of colocalization. (C) Quantitative analysis of ANGPTL4–nephrin colocalization in glomeruli from DKD and control groups. (D) Immunofluorescence staining of ANGPTL4 (green) in the tubulointerstitial compartment, with corresponding bright‑field images; double arrows highlight representative positive regions. (E) Quantification of ANGPTL4 fluorescence intensity in glomeruli and tubulointerstitium. (F) Representative images of ANGPTL4 expression across different pathological stages of DKD. (G) Comparison of mean ANGPTL4 fluorescence intensity in glomeruli and tubulointerstitium among pathological grades and between patients with or without nephrotic syndrome. (H) Schematic and representative images illustrating ANGPTL4 expression in subgroups with distinct renal injury patterns, including tubulointerstitial nephritis (TIN). (I) Comparison of ANGPTL4 expression in patients with or without TIN and with or without ischemia‑reperfusion injury (IRI). (J) Spearman correlation analyses of glomerular/tubulointerstitial ANGPTL4 levels with 24‑h urinary total protein (UTP), serum albumin (ALB), and the annual decline in estimated glomerular filtration rate (eGFR). Data are presented as mean ± SD; ns, not significant; *p < 0.05, **p < 0.01.

We next assessed the clinical relevance of this upregulation through subgroup analyses. ANGPTL4 expression did not differ across pathological grades (IIa, IIb, III) in either compartment (Figure 1F,G). However, patients with massive proteinuria exhibited significantly higher glomerular and tubulointerstitial ANGPTL4 levels than those without (Figure 1G).

Tubulointerstitial ANGPTL4 was also elevated in patients with acute/subacute tubulointerstitial nephritis (TIN, n = 12; Figure 1H,I), but was not associated with ischemia‑reperfusion injury (IRI, Figure 1I).

Furthermore, correlation analysis revealed that both glomerular and tubulointerstitial ANGPTL4 levels were associated with 24‐h urinary protein (24h UTP). Elevated tubulointerstitial ANGPTL4, which may be linked to concurrent acute/subacute TIN in some patients, was inversely associated with serum albumin and showed an association with annual estimated glomerular filtration rate (eGFR) decline (Figure 1J).

Together, these data indicate that renal ANGPTL4 expression is associated with clinical severity indices in human DKD, although further studies with larger cohorts and multivariable analyses are required to determine its independent clinical relevance.

### 2.2 Podocyte ANGPTL4 Upregulation Marks Suppressed Autophagy and Lipid Accumulation in DKD

Podocytes rely heavily on autophagy‑lysosomal degradation to maintain homeostasis [33, 34]. To define the role of ANGPTL4 within this system, we first performed gene set enrichment analysis (GSEA) on bulk RNA‐seq data (GSE30528). This revealed that ANGPTL4 expression was broadly associated with suppressed autophagy, as evidenced by significantly negative enrichment scores for multiple autophagy‐related gene sets (including macroautophagy and selective autophagy). This pattern contrasted sharply with the activation of lipid metabolism pathways (Figure 2A).

**FIGURE 2:**
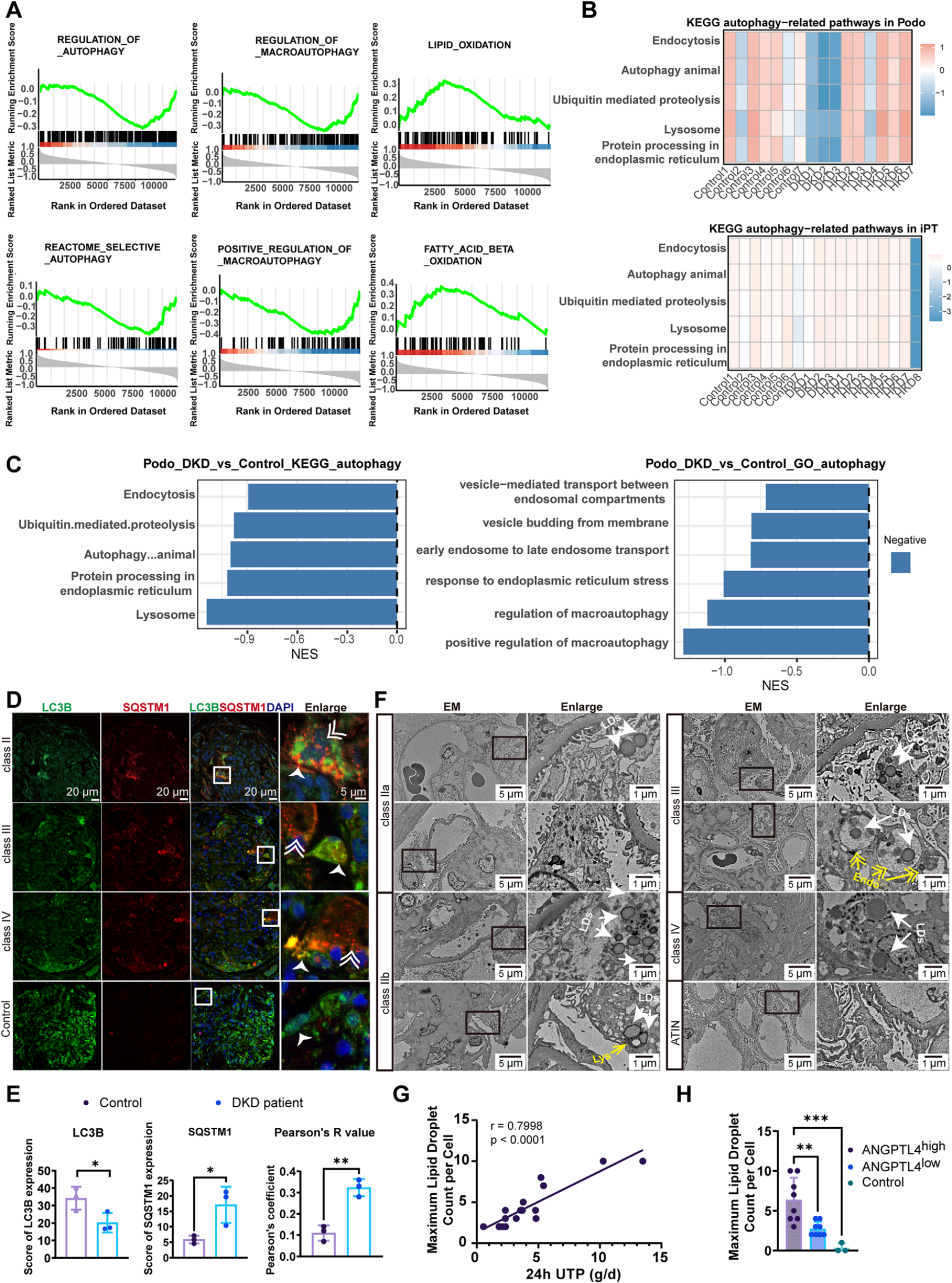
ANGPTL4 is associated with suppressed autophagy and lipid accumulation in podocytes in DKD. (A) Gene set enrichment analysis (GSEA) of the bulk RNA‑seq dataset (GSE30528) shows that ANGPTL4 expression is significantly associated with the suppression of multiple autophagy pathways and the activation of lipid oxidation‑related pathways. (B) Analysis of single‑nucleus RNA‑seq data (GSE211785) reveals downregulation of KEGG autophagy pathways specifically in podocytes, but not in tubular cells, from DKD patients compared with healthy controls and patients with hypertensive kidney disease (HKD). (C) KEGG and GO analyses further confirm an inverse correlation between ANGPTL4 expression and autophagy‑related pathways in podocytes. (D) Representative immunofluorescence images of LC3B (green) and SQSTM1/p62 (red) co‐staining in podocytes from DKD and control groups; nuclei are counterstained with DAPI (blue). Scale bar = 20 µm; insets = 5 µm. Single arrows indicate double‐positive puncta; double arrows indicate SQSTM1/p62‐only puncta. (E) Quantification of LC3B intensity, SQSTM1/p62 intensity, and LC3B‐SQSTM1/p62 colocalization in podocytes. (F) Transmission electron microscopy images showing lipid droplets and lipid‐containing autophagic/endolysosomal vesicles in podocytes from control and DKD kidney tissues. White arrowheads indicate lipid droplets. (G) Correlation between the maximum lipid‐droplet number per podocyte and 24‐h UTP. (H) Comparison of lipid‐droplet counts among control podocytes and podocytes from ANGPTL4‐low and ANGPTL4‐high DKD glomeruli. Data are presented as mean ± SD; *p < 0.05, **p < 0.01, ***p < 0.001.

To determine whether this association was cell type–specific, we performed single‐nucleus RNA sequencing (snRNA‐seq) on samples from patients with DKD (n = 3), healthy controls (n = 7), and hypertensive kidney disease (HKD, n = 8) (GSE211785). Uniform Manifold Approximation and Projection (UMAP) visualization and pathway enrichment analyses, including the Kyoto Encyclopedia of Genes and Genomes (KEGG) and Gene Ontology (GO), confirmed that autophagy was among the most significantly enriched pathways (Figure S2A,B). Cell type–specific analysis further revealed that autophagy‐related pathways were selectively suppressed in podocytes but not in tubular cells (Figure 2B; Figure S2C). Subsequent correlation analysis demonstrated a clear inverse relationship between ANGPTL4 expression and autophagy activity specifically in podocytes (Figure 2C).

We then assessed autophagic flux in renal tissues from DKD patients using immunofluorescence. Podocytes in DKD showed impaired autophagic clearance, evidenced by reduced microtubule‐associated protein 1A/1B light chain 3B (LC3B) intensity, elevated accumulation of SQSTM1/p62, and increased colocalization of LC3B with SQSTM1/p62 (Figure 2D,E). Transmission electron microscopy (TEM) provided ultrastructural evidence of autophagy–lysosome dysregulation in DKD podocytes, revealing a marked increase in lipid droplets and abundant lipid‐containing autophagic and endolysosomal vesicles (Figure 2F). Notably, the maximum number of lipid droplets per podocyte correlated positively with 24h UTP (Figure 2G).

To examine whether ANGPTL4 levels correlate with lipid load in podocytes, we stratified patients based on glomerular ANGPTL4 immunofluorescence intensity (Figure S3). Podocytes with high ANGPTL4 expression accumulated significantly more lipid droplets than those with low ANGPTL4 or control podocytes (Figure 2H), confirming that elevated ANGPTL4 is associated with greater intracellular lipid deposition.

Taken together, these findings indicate that elevated ANGPTL4 expression in DKD is associated with podocyte‐specific suppression of autophagy pathways, concomitant activation of lipid metabolism, and intracellular lipid accumulation, suggesting a potential mechanism through which ANGPTL4 may disrupt cellular homeostasis.

### 2.3 The High Glucose and Palmitic Acid (HGPA) Model Disrupts Podocyte Autophagic Flux and Induces ANGPTL4

To investigate the role of ANGPTL4 in podocyte lipotoxicity and autophagy, we first screened multiple DKD models for lipid droplet accumulation in podocytes using high‑resolution electron microscopy. In streptozotocin/high‐fat diet (STZ/HFD)‐induced diabetic rats, lipid droplets accumulated markedly in tubular cells but were nearly absent in podocytes even after four months (Figure S4A). Similarly, in the genetic diabetic db/db mouse model—a canonical model of type 2 diabetes and DKD—we observed characteristic podocyte injuries, including glomerular basement membrane thickening and foot process effacement at both 16 and 28 weeks of age. However, despite these pronounced pathological changes, podocyte lipid droplets were notably absent in *db/db* mice at both time points (Figure S4B). In contrast, as a positive control, the adriamycin (ADR) mouse model showed prominent lipid droplet accumulation in podocytes as early as one week post‑injection (Figure S4A). This model‐specific disparity highlighted the need for a complementary in vitro system to reliably study podocyte lipotoxicity. In immortalized human podocytes (HPCs), we compared several treatments—high glucose (HG), advanced glycation end‐product‐modified bovine serum albumin (AGE‑BSA), and HG plus palmitic acid (HGPA). Among these, HGPA most effectively induced substantial lipid droplet formation (Figure 3A) and was therefore selected for subsequent studies.

**FIGURE 3:**
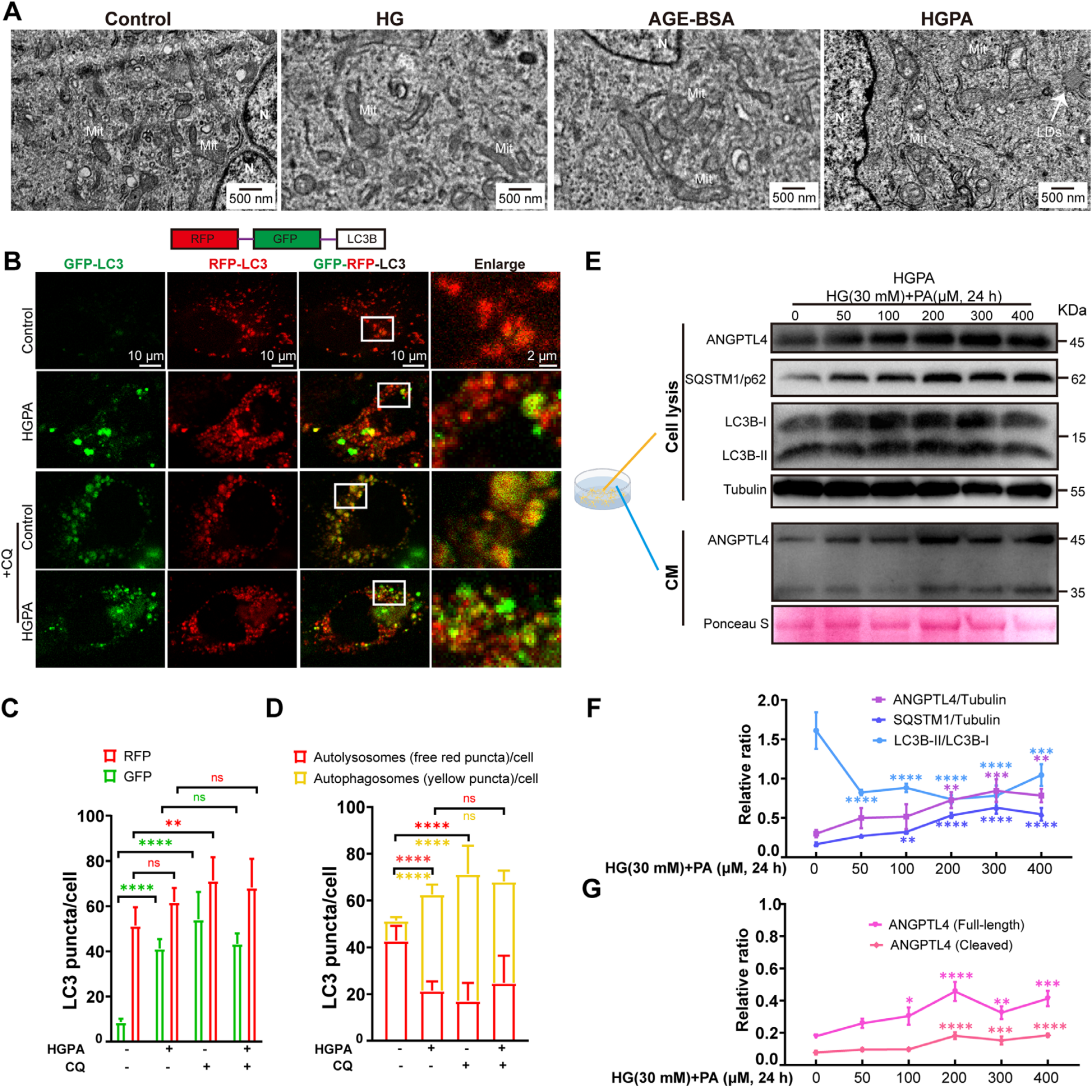
HGPA induces autophagic dysfunction and PA dose‑dependently upregulates ANGPTL4 in podocytes. (A) Transmission electron microscopy images showing lipid droplets (white arrows) in serum‑starved human podocytes (HPCs) cultured under control, high glucose (HG), AGE‑BSA, or HGPA conditions. Scale bar = 500 nm. (B‐‑D) HPCs expressing the RFP‑GFP‑LC3B reporter were treated with control or HGPA medium, with or without chloroquine (CQ). (B) Representative fluorescence images and quantification of GFP and RFP puncta per cell. (C) Numbers of autophagosomes (yellow puncta in merged images) and autolysosomes (red‑only puncta) per cell. (D) Schematic summary of autophagic flux under the indicated conditions. (E‐‑G) Serum‑starved HPCs were treated with HG (30 mM) and increasing concentrations of palmitic acid (PA; 0–400 µM) for 24 h. (E) Western blot analysis of full‑length ANGPTL4, SQSTM1/p62, LC3B, and tubulin in cell lysates, and of full‑length and cleaved ANGPTL4 in conditioned media; Ponceau S staining serves as the loading control for secreted proteins. (F) Quantification of full‑length ANGPTL4/tubulin, SQSTM1/p62/tubulin, and LC3B‑II/LC3B‑I ratios in cell lysates. (G) Secreted levels of full‑length and cleaved ANGPTL4 in conditioned media, normalized to total protein assessed by Ponceau S staining. Data are presented as mean ± SD (n ≥ 3). ns, not significant; *p < 0.05, **p < 0.01, ***p < 0.001, ****p < 0.0001.

Using the RFP–GFP–LC3B tandem reporter system, we assessed autophagic flux under HGPA conditions. HGPA treatment significantly increased GFP‑LC3 puncta without altering RFP‑LC3 puncta (Figure 3B–D). In control cells, chloroquine (CQ) induced the expected blockade of autophagic flux, accumulating both GFP‑LC3 and RFP‑LC3. In HGPA‑treated cells, however, CQ caused no further accumulation of either reporter, indicating concurrent impairment of autophagosome formation and autophagic flux. Quantitative analysis confirmed that HGPA reduced autolysosome numbers while increasing autophagosome accumulation, phenocopying the effect of CQ (Figure 3D). Subsequent CQ treatment after HGPA did not significantly alter autophagosome or autolysosome levels.

We further evaluated whether HGPA regulates ANGPTL4 expression. Podocytes were treated with increasing concentrations of PA (50–400 µM) plus HG (30 mM) for 24 h (Figure 3E). Western blot of cell lysates (Figure 3F) showed concentration‑dependent upregulation of ANGPTL4 (200–400 µM), accompanied by p62 accumulation (100–400 µM) and a decreased LC3B‑II/LC3B‑I ratio (50–400 µM). In conditioned medium, both the 45 kDa full‑length and 35 kDa cleaved forms of ANGPTL4 were elevated in a PA‑dependent manner, with the full‑length form significantly increased at 100 µM (Figure 3G).

Together, these results establish HGPA as a robust model of podocyte lipotoxicity that concurrently disrupts autophagic flux and upregulates ANGPTL4 expression.

### 2.4 ANGPTL4 Impairs Podocyte Lipophagy and Promotes Lipid Accumulation

To examine the role of ANGPTL4 in podocyte lipophagy, we overexpressed ANGPTL4 in HPCs and performed live‑cell imaging using LC3B‑RFP (autophagosomes) and BODIPY  493/503 (lipid droplets), followed by quantitative analysis of lipid‑droplet parameters and their colocalization with autophagosomes; results were further validated by electron microscopy (Figure 4A,B).

**FIGURE 4:**
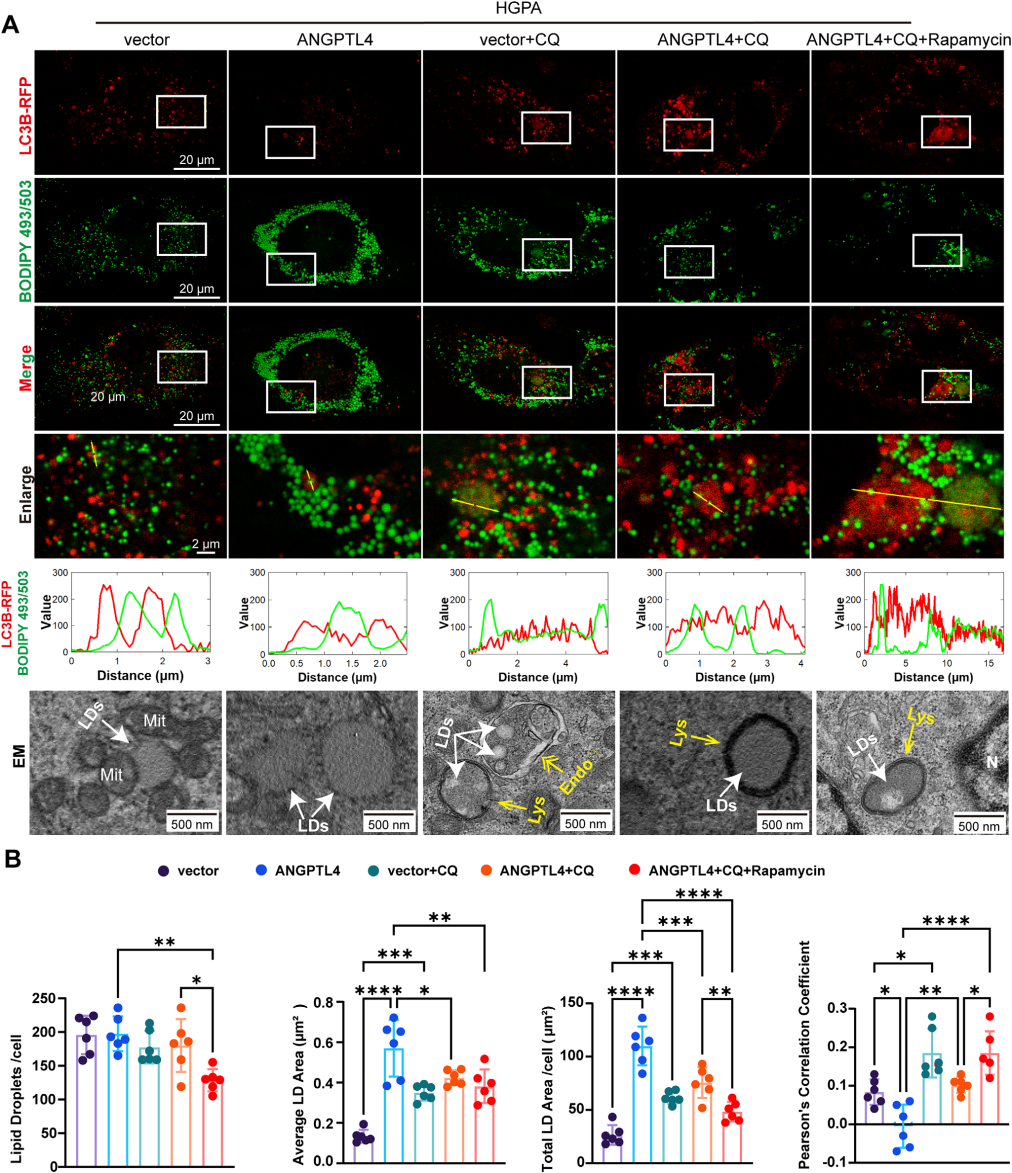
ANGPTL4 overexpression impairs lipophagic flux and promotes lipid accumulation in podocytes under diabetic conditions. (A) Podocytes were transfected with an ANGPTL4‑overexpressing plasmid or empty vector for 72 h, followed by HGPA treatment. Cells were then subjected to the indicated interventions: chloroquine (CQ, 50 µM, 4 h) or rapamycin (Rapa, 100 nM, 6 h) + CQ (50 µM, 4 h). Prior to live‑cell imaging, cells were transfected with LC3B‑RFP to label autophagosomes and stained with BODIPY 493/503 to visualize lipid droplets. Upper panels: confocal micrographs (scale bar = 20 µm; inset = 2 µm). Middle panels: fluorescence intensity profiles along the yellow line in the enlarged regions, showing the colocalization of autophagosomes (LC3B‑RFP, red) and lipid droplets (BODIPY, green). Lower panels: transmission electron microscopy images (scale bar = 500 nm). White arrows: lipid droplets (LDs); yellow double arrows: early endosomes; yellow arrows: lysosomes (Lys). (B) Quantitative analysis of lipid droplet parameters and autophagosome‑lipid droplet colocalization across the five experimental groups. From left to right: number of lipid droplets per cell, average lipid droplet area per cell, total lipid droplet area per cell, and Pearson's colocalization coefficient between LC3B‑RFP and BODIPY 493/503. Data are presented as mean ± SD; *p < 0.05, **p < 0.01, ***p < 0.001, ****p < 0.0001.

Quantitative analysis showed that ANGPTL4 overexpression did not significantly increase lipid‑droplet number but markedly elevated both average droplet size and total lipid area per cell compared with vector controls (Figure 4A,B). In control cells, chloroquine (CQ) treatment significantly increased colocalization between lipid droplets and autophagosomes. Plot‑profile analysis indicated that lipid droplets were engulfed by autophagosomes, with fluorescence intensity reduced by approximately half relative to free droplets. Transmission electron microscopy provided ultrastructural evidence of multiple small lipid droplets enclosed within autophagosomes and endosomes.

In ANGPTL4‑overexpressing cells, however, autophagosome‑lipid droplet colocalization was even lower, with some cases showing negative coefficients. Plot profiles revealed that although lipid droplets were surrounded by autophagosomal structures, their fluorescence intensity remained similar to that of free droplets. CQ treatment increased colocalization but did not reduce the fluorescence intensity of engulfed lipid droplets. EM images displayed lipid droplets trapped within lysosomes. Rapamycin treatment enhanced colocalization and partially reduced the fluorescence intensity of encapsulated lipid droplets, accompanied by decreases in lipid‑droplet number, average size, and total area compared with ANGPTL4 overexpression alone.

Together, these data demonstrate that ANGPTL4 overexpression leads to accumulation of undegraded lipid droplets, indicating a specific impairment in lysosomal degradation during lipophagy.

### 2.5 ANGPTL4 Disrupts Podocyte Autophagic Flux and Lysosomal Function

Having demonstrated that ANGPTL4 blocks lipophagic flux, we next asked whether it also disrupts general autophagic progression and lysosomal function. To this end, we treated ANGPLT4‑overexpressing podocytes with bafilomycin A1 (Baf A1) or rapamycin and assessed key autophagic markers (Figure 5A,B). Immunofluorescence quantification revealed that ANGPTL4 overexpression significantly increased total p62 accumulation without altering its colocalization with LC3B. In vector‑control cells, Baf A1 induced pronounced p62 accumulation together with increased LC3B‑colocalized p62, whereas rapamycin reduced both total p62 and its colocalization with LC3B. In contrast, ANGPTL4‑overexpressing cells exhibited enhanced sensitivity to Baf A1, showing stronger p62 accumulation and elevated LC3B‑colocalized p62. Moreover, these cells were resistant to rapamycin, as neither total p62 nor LC3B colocalization changed significantly.

**FIGURE 5:**
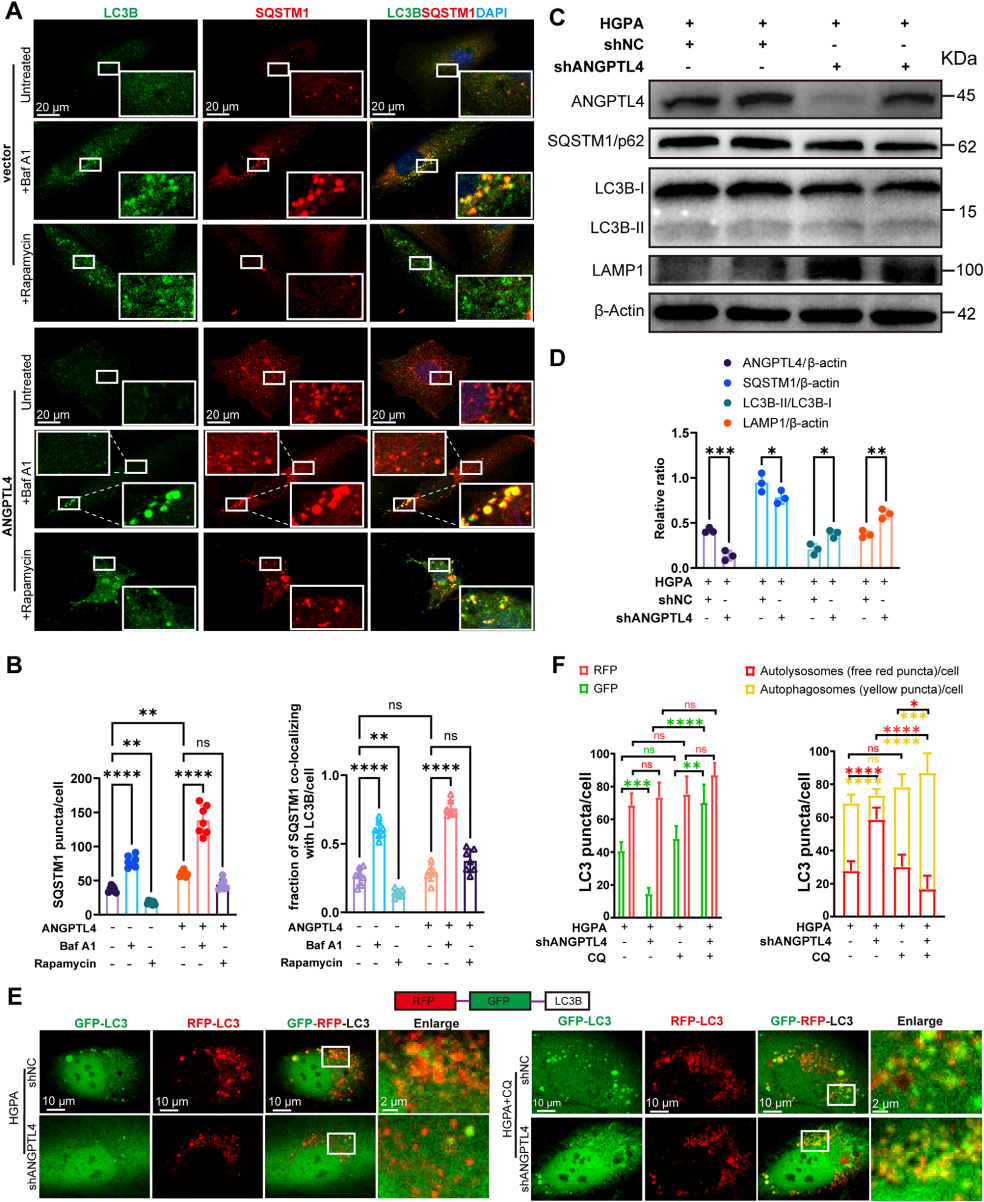
ANGPTL4 impairs autophagic flux and lysosomal function in podocytes. (A) Schematic of experimental treatments and representative immunofluorescence images. Podocytes were transfected with ANGPTL4‑overexpressing plasmid or empty vector for 72 h, followed by transfection with LC3B‑RFP. Cells were then treated with bafilomycin A1 (Baf A1, 100 nM, 4 h) or rapamycin (100 nM, 6 h). After fixation, cells were immunostained for LC3B (green) and SQSTM1/p62 (p62, red); nuclei were stained with DAPI (blue). Scale bar = 20 µm. (B) Quantification of total p62 puncta per cell (left) and the fraction of p62 colocalizing with LC3B (right) across six experimental groups. Data are mean ± SD (n ≥ 3). (C, D) ANGPTL4 knockdown rescues autophagy under HGPA stress. (C) Western blot analysis of ANGPTL4, SQSTM1/p62, LC3B, LAMP1, and β‑actin in podocytes transduced with scramble (shNC) or ANGPTL4‑targeting (shANGPTL4) shRNA followed by HGPA treatment. (D) Quantification of ANGPTL4/β‑actin, SQSTM1/β‑actin, and LC3B‑II/LC3B‑I ratios. Data are mean ± SD (n ≥ 3). (E, F) ANGPTL4 knockdown restores functional autophagic flux. (E) Representative confocal images of podocytes expressing the RFP‑GFP‑LC3B reporter after the indicated treatments. Yellow puncta (RFP+GFP+) represent autophagosomes; red‑only puncta (RFP+GFP−) represent autolysosomes. (F) Quantification of red and yellow puncta per cell (left) and the numbers of autophagosomes and autolysosomes per cell (right). Data are mean ± SD (n ≥ 3). Statistical significance: *p < 0.05, **p < 0.01, ***p < 0.001, ****p < 0.0001; ns, not significant.

To further examine the role of ANGPTL4 under diabetic conditions, we knocked down ANGPTL4 and treated cells with HGPA. Western blot analysis confirmed that ANGPTL4 knockdown reduced p62 accumulation while increasing the LC3B‑II/LC3B‑I ratio and lysosome‐associated membrane protein 1 (LAMP1) expression (Figure 5C,D). Using the RFP‑GFP‑LC3B reporter system, we found that shANGPTL4 cells displayed unchanged RFP‑LC3B puncta but significantly fewer GFP‑LC3B puncta, along with increased autolysosome and autophagosome numbers (Figure 5E,F). In control cells, CQ induced only minor changes in GFP‑LC3B, RFP‑LC3B, autolysosomes, or autophagosomes. In shANGPTL4 cells, however, CQ triggered marked increases in GFP‑LC3B puncta, autolysosomes, and autophagosomes.

Together, these results demonstrate that ANGPTL4 disrupts autophagic termination and lysosomal degradation, whereas its knockdown enhances autophagic clearance under diabetic conditions, identifying ANGPTL4 as a key regulator of podocyte lipotoxicity.

### 2.6 ANGPTL4 Impairs TFEB Nuclear Translocation and Lysosomal Degradative Function

To define the molecular pathways regulated by ANGPTL4 under diabetic conditions, we performed RNA sequencing in HGPA‐treated podocytes following ANGPTL4 knockdown. Comparison of shANGPTL4 + HGPA podocytes with shNC + HGPA controls revealed broad alterations in genes associated with autophagy, lysosomal function, and lipid metabolism (Figure 6A). A focused heatmap further showed coordinated upregulation of *TFEB*, *MAP1LC3B*, and *LIPA*, together with reduced *PNPLA3* expression following ANGPTL4 knockdown (Figure 6B).

**FIGURE 6:**
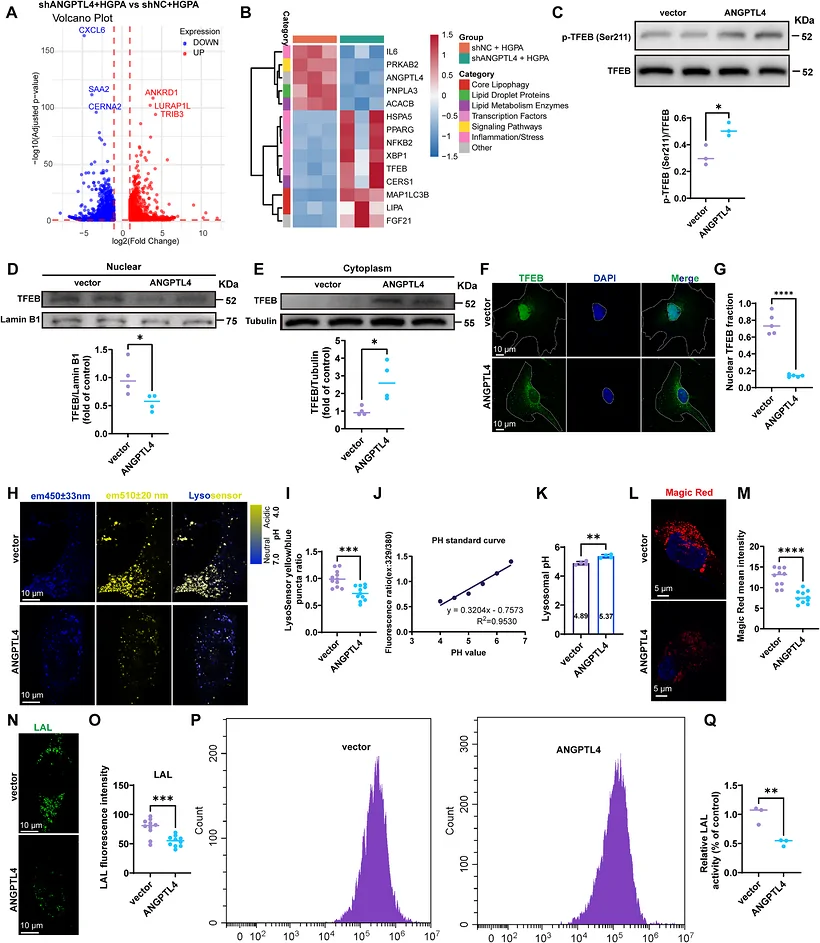
ANGPTL4 overexpression impairs TFEB nuclear translocation and lysosomal function in podocytes. (A) Volcano plot of differentially expressed genes in podocytes treated with shANGPTL4 versus shNC under HGPA conditions (∣log_2_FC∣ > 1, padj < 0.05). Red and blue dots indicate significantly up‑ and down‑regulated genes, respectively. Top five genes in each direction are labeled. (B) Heatmap showing expression patterns of selected autophagy‑ and lysosome‑related genes in shANGPTL4+HGPA versus shNC+HGPA groups (n = 3 per group). (C) Representative immunoblots and quantitative analysis of TFEB phosphorylation at Ser211 in vector‐control and ANGPTL4‐overexpressing podocytes. Phosphorylated TFEB was normalized to total TFEB (n = 3 independent experiments). (D, E) Representative immunoblots and quantitative analyses of TFEB protein levels in the nuclear (D) and cytoplasmic (E) fractions of vector‐control and ANGPTL4‐overexpressing podocytes. Lamin B1 and tubulin were used as loading controls for the nuclear and cytoplasmic fractions, respectively (n = 4). (F) Representative immunofluorescence images of TFEB localization in vector‐control and ANGPTL4‐overexpressing podocytes. TFEB is shown in green, nuclei were counterstained with DAPI (blue), and merged images are shown. Cell and nuclear boundaries are outlined in white. Scale bars, 10 µm. (G) Quantification of the percentage of TFEB signal localized within the nucleus in the indicated groups (n = 5 cells per group). (H) Representative images of podocytes stained with LysoSensor Yellow/Blue DND‐160. Fluorescence was detected in the blue emission channel (450 ± 33 nm) and yellow emission channel (510 ± 20 nm), and merged images are shown. Images were acquired using a Leica STELLARIS 5 confocal microscope. Scale bars, 10 µm. (I) Quantification of the LysoSensor yellow‐to‐blue fluorescence ratio in vector‐control and ANGPTL4‐overexpressing podocytes (n = 11 cells per group). (J) Calibration curve showing the relationship between the LysoSensor fluorescence ratio and the pH of standard calibration buffers. (K) Lysosomal pH values in vector‐control and ANGPTL4‐overexpressing podocytes calculated from the calibration curve shown in J (n = 4 replicate wells per group). (L) Representative fluorescence images of cathepsin B activity detected using the Magic Red cathepsin B substrate in vector‐control and ANGPTL4‐overexpressing podocytes. Images were acquired using a Leica STELLARIS 5 confocal microscope. Scale bars, 5 µm. (M) Quantification of Magic Red mean fluorescence intensity (n = 10 cells per group). (N) Representative fluorescence images of lysosomal acid lipase (LAL) activity detected using the LipaGreen probe in vector‐control and ANGPTL4‐overexpressing podocytes. Images were acquired using a Leica STELLARIS 5 confocal microscope. Scale bars, 10 µm. (O) Quantification of LipaGreen fluorescence intensity (n = 10 cells per group). (P) Representative flow‐cytometry histograms of LipaGreen fluorescence in vector‐control and ANGPTL4‐overexpressing podocytes. (Q) Quantification of relative LAL activity determined by flow cytometry (n = 3 independent experiments). For the cell‐based imaging analyses in G, I, M, and O, cells were pooled from three independent experiments. Data are presented as the mean ± SD. Statistical significance: *p < 0.05, **p < 0.01, ***p < 0.001, ****p < 0.0001; ns, not significant.

Given the established role of TFEB in lysosomal biogenesis and autophagy, we next examined whether ANGPTL4 affects TFEB activation and subcellular localization. ANGPTL4 overexpression was associated with increased TFEB phosphorylation at Ser211, as reflected by an elevated p‐TFEB (Ser211)/total TFEB ratio (Figure 6C). Nuclear–cytoplasmic fractionation followed by immunoblotting showed that ANGPTL4 overexpression markedly reduced nuclear TFEB while increasing its cytoplasmic abundance (Figure 6D,E). Immunofluorescence analysis further confirmed a significant reduction in TFEB nuclear localization in ANGPTL4‐overexpressing podocytes (Figure 6F,G). These findings indicate that ANGPTL4 impairs TFEB nuclear translocation in podocytes.

Given the impaired lipophagic flux observed in ANGPTL4‐overexpressing podocytes, we next investigated whether this defect was associated with compromised lysosomal acidification and degradative function. Confocal imaging with LysoSensor Yellow/Blue DND‐160 showed that ANGPTL4 overexpression reduced the yellow‐to‐blue fluorescence ratio by approximately 30% compared with vector controls, indicating diminished lysosomal acidification (Figure 6H,I). To obtain absolute measurement, fluorescence ratios were subsequently determined using a microplate reader and converted to lysosomal pH values using a calibration curve. ANGPTL4 overexpression increased lysosomal pH from 4.89 to 5.37, corresponding to an elevation of 0.48 pH units and confirming defective lysosomal acidification (Figure 6J,K).

As lysosomal hydrolase activity depends on an acidic luminal environment, we next examined whether impaired acidification was accompanied by reduced degradative enzyme activity. Cathepsin B was assessed as a representative lysosomal protease reflecting general proteolytic capacity, whereas lysosomal acid lipase (LAL) was selected for its direct role in lysosomal lipid hydrolysis during lipophagy. *LIPA*, which encodes LAL, was significantly upregulated following ANGPTL4 knockdown (Figure 6B). Consistent with the acidification defect, Magic Red staining showed reduced cathepsin B activity in ANGPTL4‐overexpressing podocytes (Figure 6L,M). LAL activity, assessed using the LipaGreen probe by confocal imaging and flow cytometry, was likewise significantly diminished (Figure 6N–Q).

Together, these findings demonstrate that ANGPTL4 impairs TFEB nuclear translocation and disrupts lysosomal degradative function by compromising acidification, proteolytic activity, and lysosomal lipid hydrolysis, thereby providing a mechanistic basis for defective podocyte lipophagy.

### 2.7 Extracellular ANGPTL4 Impairs Podocyte Lipophagy

Given the secreted nature of ANGPTL4 and its detectable presence in podocyte‐conditioned medium, we next investigated whether extracellular ANGPTL4 could recapitulate the lysosomal and lipophagic defects observed in ANGPTL4‐overexpressing podocytes. To determine whether extracellular ANGPTL4 engages the TFEB–lysosome axis, we first examined the subcellular distribution of TFEB. Treatment with recombinant human N‐terminal ANGPTL4 fragment (rhANGPTL4) markedly reduced nuclear TFEB while increasing its cytoplasmic abundance. Neutralization with an ANGPTL4‐neutralizing antibody partially restored TFEB nuclear localization, while expression of the constitutively active TFEB‐S211A mutant produced a more pronounced rescue (Figure 7A,B).

**FIGURE 7:**
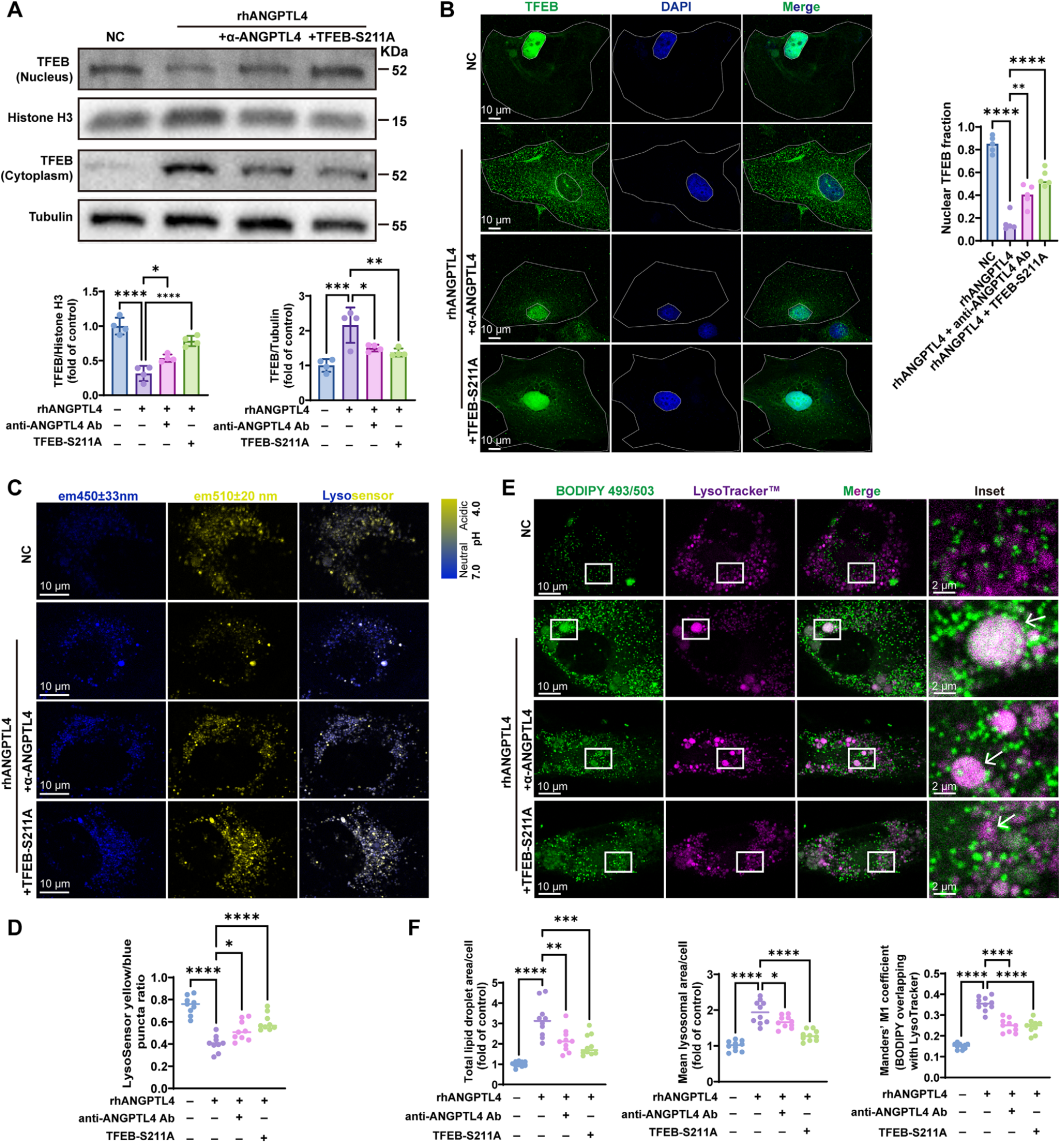
Recombinant N‐terminal ANGPTL4 fragment impairs podocyte lipophagy. (A) Representative immunoblots and quantitative analyses of TFEB protein levels in the nuclear and cytoplasmic fractions of podocytes treated with vehicle (NC), recombinant human N‐terminal ANGPTL4 fragment (rhANGPTL4), rhANGPTL4 plus an ANGPTL4‐neutralizing antibody (α‐ANGPTL4 Ab), or rhANGPTL4 with TFEB‐S211A expression. Histone H3 and tubulin were used as loading controls for the nuclear and cytoplasmic fractions, respectively (n = 4 independent experiments). (B) Representative immunofluorescence images and quantification of TFEB nuclear localization in the indicated groups. TFEB is shown in green, nuclei were counterstained with DAPI (blue), and merged images are shown. Images were acquired using a Leica STELLARIS 5 confocal microscope. Cell and nuclear boundaries are outlined in white. Scale bars, 10 µm. Each dot represents one cell. (C) Representative images of podocytes stained with LysoSensor Yellow/Blue DND‐160. Fluorescence was detected in the blue emission channel (450 ± 33 nm) and yellow emission channel (510 ± 20 nm), and merged images are shown. Images were acquired using a Leica STELLARIS 5 confocal microscope. Scale bars, 10 µm. (D) Quantification of the LysoSensor yellow‐to‐blue fluorescence ratio in podocytes treated with vehicle (NC), rhANGPTL4, rhANGPTL4 plus an ANGPTL4‐neutralizing antibody, or rhANGPTL4 with TFEB‐S211A expression. Each dot represents one cell; cells were pooled from three independent experiments. (E) Representative fluorescence images of lipid droplets stained with BODIPY 493/503 (green) and lysosomes stained with LysoTracker (magenta) in the indicated groups. Merged images and enlarged views of the boxed regions are shown. Arrows indicate colocalization of lipid droplets with lysosomes. Images were acquired using a Leica STELLARIS 5 confocal microscope. Scale bars, 10 µm; insets, 2 µm. (F) Quantification of the total lipid‐droplet area per cell, mean lysosomal area per cell, and Manders’ M1 coefficient representing the fraction of BODIPY‐positive signal overlapping with LysoTracker‐positive signal. Values for lipid‐droplet and lysosomal areas were normalized to the NC group. Each dot represents one cell. Cells used for imaging‐based analyses were pooled from three independent experiments. Data are presented as the mean ± SD. Statistical significance: *p < 0.05, **p < 0.01, ***p < 0.001, ****p < 0.0001; ns, not significant.

We next assessed the functional effects of extracellular ANGPTL4 on lysosomal acidification. rhANGPTL4 treatment significantly reduced the LysoSensor yellow‐to‐blue fluorescence ratio, indicating impaired lysosomal acidification. Neutralization of extracellular ANGPTL4 and expression of TFEB‐S211A both restored the fluorescence ratio, with TFEB‐S211A producing a more pronounced rescue (Figure 7C,D). These findings indicate that extracellular ANGPTL4 impairs lysosomal acidification and support the involvement of TFEB in mediating this effect.

We then examined whether extracellular ANGPTL4‐induced lysosomal dysfunction was accompanied by impaired lipophagic clearance. Lipid droplets and lysosomes were visualized by co‐staining with BODIPY 493/503 and LysoTracker, respectively. rhANGPTL4 treatment increased the total lipid‐droplet area and mean lysosomal area per cell, together with an increased fraction of BODIPY‐positive signal overlapping with LysoTracker‐positive signal. In the context of impaired lysosomal acidification, these changes indicate the retention of lipid droplets within dysfunctional lysosomal compartments. Both ANGPTL4 neutralization and TFEB‐S211A expression attenuated lipid‐droplet accumulation, lysosomal enlargement, and BODIPY–LysoTracker colocalization (Figure 7E,F).

Collectively, these findings demonstrate that recombinant human N‐terminal ANGPTL4 fragment is sufficient to reproduce several TFEB‐associated lysosomal and lipophagic defects. The rescue achieved by ANGPTL4 neutralization and TFEB‐S211A expression supports a functional role for secreted ANGPTL4 acting through TFEB, although an additional intracellular contribution cannot be excluded.

### 2.8 ANGPTL4 Promotes Podocyte Fibrotic Remodeling

Progressive renal fibrosis is a major pathological feature of DKD and a key determinant of disease progression. We therefore examined whether ANGPTL4‐associated impairment of the autophagy–lysosome system was linked to profibrotic remodeling in podocytes. In renal biopsy specimens, glomeruli with high ANGPTL4 expression exhibited markedly higher levels of collagen I, α‐smooth muscle actin, fibronectin, and collagen III than ANGPTL4‐low glomeruli and controls (Figure 8A,B). Pathway activity analysis further showed that ANGPTL4 knockdown under HGPA conditions markedly increased the autophagy activity score, while reducing the ANGPTL4 and epithelial–mesenchymal transition (EMT) activity scores compared with the shNC + HGPA group (Figure 8C). In cultured podocytes, ANGPTL4 overexpression increased SQSTM1/p62 accumulation, reduced the LC3B‐II/LC3B‐I ratio and synaptopodin expression, and increased COL3A1 abundance, indicating impaired autophagic clearance, loss of the differentiated podocyte phenotype, and enhanced extracellular matrix production (Figure 8D,E).

**FIGURE 8:**
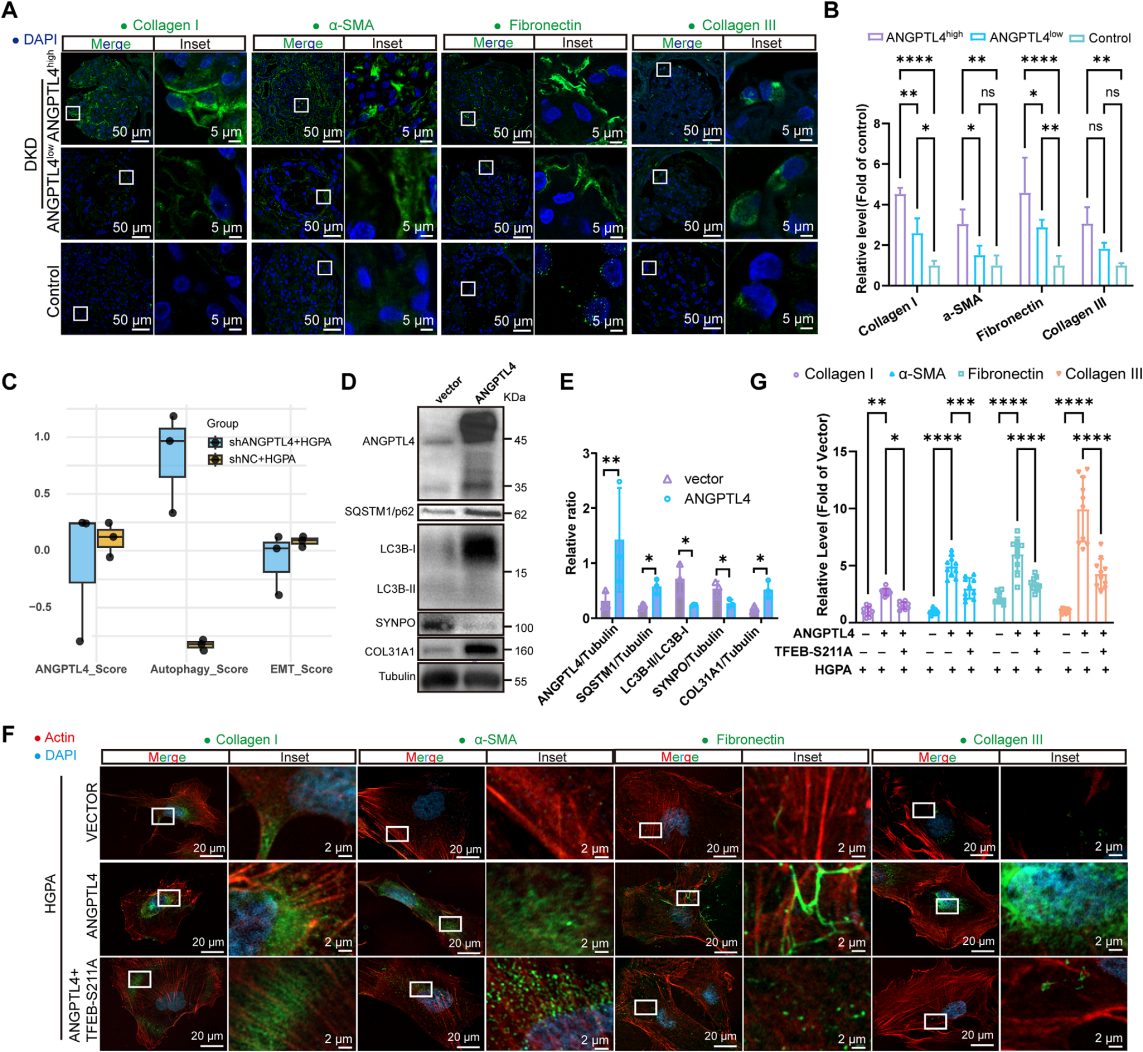
ANGPTL4 promotes podocyte fibrotic remodeling. (A) Representative immunofluorescence images of collagen I, α‐smooth muscle actin, fibronectin, and collagen III in kidney biopsy specimens from patients with diabetic kidney disease (DKD) exhibiting high or low glomerular ANGPTL4 expression and from control subjects. The indicated extracellular matrix and profibrotic markers are shown in green, and nuclei were counterstained with DAPI (blue). Boxed regions are shown at higher magnification. Scale bars, 50 µm; insets, 5 µm. (B) Quantification of collagen I, α‐SMA, fibronectin, and collagen III fluorescence levels in the ANGPTL4^high^, ANGPTL4low, and control groups. Values were normalized to those of the control group. (C) Comparison of ANGPTL4, autophagy, and epithelial–mesenchymal transition (EMT) pathway activity scores between the shANGPTL4 + HGPA and shNC + HGPA groups (n = 3 per group). (D) Representative immunoblots of ANGPTL4, SQSTM1/p62, LC3B‐I/II, synaptopodin (SYNPO), and collagen III α1 chain (COL3A1) in vector‐control and ANGPTL4‐overexpressing podocytes. Tubulin was used as the loading control. (E) Densitometric quantification of ANGPTL4, SQSTM1/p62, the LC3B‐II/LC3B‐I ratio, SYNPO, and COL3A1 in the indicated groups. Protein levels were normalized to tubulin, except for LC3B, which is expressed as the LC3B‐II/LC3B‐I ratio. (F) Representative immunofluorescence images of collagen I, α‐SMA, fibronectin, and collagen III in HGPA‐treated podocytes transfected with vector, ANGPTL4, or ANGPTL4 together with constitutively active TFEB‐S211A. The indicated profibrotic markers are shown in green, F‐actin was stained with phalloidin (red), and nuclei were counterstained with DAPI (blue). Boxed regions are shown at higher magnification. Scale bars, 20 µm; insets, 2 µm. (G) Quantification of collagen I, α‐SMA, fibronectin, and collagen III fluorescence levels in the indicated groups. Values were normalized to the HGPA‐treated vector‐control group. Data are presented as the mean ± SD. Statistical significance: *p < 0.05, **p < 0.01, ***p < 0.001, ****p < 0.0001; ns, not significant.

To further examine whether TFEB mediates ANGPTL4‐induced profibrotic remodeling under diabetic stress, we evaluated these markers in HGPA‐treated podocytes. ANGPTL4 overexpression further increased the levels of collagen I, α‐SMA, fibronectin, and collagen III, whereas co‐expression of TFEB‐S211A markedly attenuated the accumulation of all four markers (Figure 8F,G).

Collectively, these findings support that ANGPTL4 promotes podocyte profibrotic remodeling, at least in part, through suppression of TFEB‐mediated autophagic and lysosomal function.

## 3 Discussion

DKD progression is driven by podocyte injury and dysregulated renal lipid metabolism; however, the molecular links that translate diabetic metabolic stress into cellular dysfunction remain incompletely understood. Through an integrative approach combining human tissue analyses with functional validation in podocytes, our data support ANGPTL4 as an important modulator associated with DKD pathology. The in vitro findings support a model in which diabetic metabolic stress increases ANGPTL4 expression, impairs TFEB‐associated lysosomal function, and disrupts lipophagy, thereby promoting podocyte lipotoxicity.

Analysis of human renal biopsies revealed significant upregulation of ANGPTL4 in both glomerular podocytes and tubular epithelial cells in DKD. This finding extends recent animal studies and supports a disease‐relevant role for renal ANGPTL4 in DKD [31]. Importantly, its expression pattern appears to be disease‐context specific. While ANGPTL4 levels rapidly normalize after treatment in primary nephrotic syndrome, sustained ANGPTL4 overexpression in DKD was associated with persistent proteinuria and more rapid renal functional decline, suggesting that chronic hyperglycemic and lipotoxic stress may perpetuate ANGPTL4 dysregulation [29]. Recent studies further suggest that ANGPTL4 derived from both glomerular and tubular compartments may promote renal profibrotic remodeling, indicating that its pathogenic effects in DKD extend beyond podocytes [28]. These findings support a broader, cell type‐dependent role for ANGPTL4 within the renal microenvironment.

Lipid metabolism and autophagy are complex, dynamically regulated processes with considerable mechanistic redundancy [35, 36]. Podocytes, which have high energy demands and are susceptible to shear stress, depend heavily on functional autophagy and lysosomal degradation to maintain cellular homeostasis and glomerular integrity [18, 37, 38, 39, 40, 41, 42, 43, 44]. This reliance on precise lipid clearance and organelle function is part of a broader paradigm in which cellular metabolism—particularly cholesterol and lipid homeostasis—directly governs cell survival, identity, and adaptive responses [45]. Consistent with this, our bioinformatic analysis indicates that autophagic function is more severely impaired in DKD podocytes than in renal tubular cells. This finding aligns with prior reports connecting podocyte injury to autophagy dysfunction and supports the concept of suppressed renal autophagic activity in early diabetes [41, 46, 47, 48, 49]. Although dysregulated podocyte lipid metabolism is increasingly linked to nephropathy, the precise role of lipotoxicity in DKD podocytes remains unclear [50, 51]. This knowledge gap stems partly from a key translational challenge: whereas prominent podocyte lipid droplet accumulation is a recognized feature of human DKD biopsies, conventional rodent models (e.g., *db/db* mice, STZ‐induced diabetes) primarily show lipid deposition in tubular cells, with inconsistent or minimal podocyte lipidosis [52, 53]. This renal tubule‐predominant lipid deposition is corroborated by multiple independent studies. For instance, Oil Red O or Nile red staining in db/db and HFD/STZ‐induced diabetic mice consistently reveals abundant lipid droplets specifically in the tubular compartments of the kidney [54, 55, 56]. Our electron microscopy analysis likewise showed that db/db mice developed typical diabetic glomerular injury but lacked detectable lipid droplets in podocytes at both 16 and 28 weeks of age. This discrepancy between human DKD and conventional diabetic rodent models may reflect species‐ and model‐dependent differences in podocyte lipid uptake, storage, utilization, and stress responses, as well as differences in the duration and severity of metabolic exposure and the kinetics of disease progression. In addition, diabetic rodent kidneys commonly exhibit predominantly tubular lipid deposition, whereas prominent podocyte lipid accumulation is more consistently observed in human DKD. These limitations indicate that conventional diabetic rodent models may not fully reproduce the podocyte lipid‐metabolic phenotype of human DKD and support the complementary use of human kidney tissue and HGPA‐treated human podocytes for mechanistic studies.

Pathological lipid deposition in podocytes is increasingly attributed to dysregulation of intracellular lipid metabolism—encompassing synthesis, uptake, storage, utilization, and efflux—rather than solely mirroring systemic lipid levels [52, 57]. Beyond the diabetic milieu, dysregulated intracellular lipid metabolism is a hallmark of other major chronic diseases. For instance, in cardiovascular pathologies, research has shown that specific pathogenic stimuli can directly trigger profound lipid droplet accumulation and a profibrotic phenotypic shift in relevant parenchymal cells, independent of classical circulating lipid overload [58]. Here, we focused on a critical component of this regulatory network: lipophagy, the selective autophagic degradation of lipid droplets [59, 60, 61]. To overcome the limitations of animal models in reproducing human podocyte lipotoxicity, we used an in vitro HGPA model that recapitulates diabetic lipotoxic stress and induces neutral lipid deposition. HGPA markedly upregulated ANGPTL4 expression, and ANGPTL4 overexpression promoted substantial lipid droplet accumulation. Given the dependence of podocytes on efficient lipophagy for lipid homeostasis, we examined this pathway as a potential target of ANGPTL4. ANGPTL4 overexpression markedly reduced lipid droplet–autophagosome colocalization, while electron microscopy revealed lipid droplets retained within undegraded lysosomal compartments. The partial reversal of these abnormalities by rapamycin further supported impaired lipophagic flux. These findings indicate that ANGPTL4 promotes podocyte lipotoxicity by disrupting an essential intracellular lipid‐clearance pathway. Consistent with broader lipid metabolic reprogramming, ANGPTL4 knockdown under HGPA conditions increased PNPLA2 expression and decreased PNPLA3 expression. As PNPLA2 contains an LC3‐interacting region that may facilitate lipid droplet recruitment to autophagosomes, these transcriptional changes may also favor lipophagy initiation [62, 63].

Our findings further identify impaired lysosomal competence as a mechanistic basis for the ANGPTL4‐induced lipophagic blockade. TFEB coordinates the transcriptional program governing lysosomal biogenesis, autophagy, and cellular clearance, and its nuclear localization is required for transcriptional activation of the CLEAR network [64, 65]. In our study, ANGPTL4 knockdown increased TFEB expression, whereas ANGPTL4 overexpression reduced nuclear TFEB and promoted its cytoplasmic retention. These findings suggest that ANGPTL4 suppresses the TFEB‐dependent lysosomal adaptive response. The ability of TFEB‐S211A, a mutant with enhanced nuclear localization and transcriptional activity, to restore lysosomal function and lipid clearance further implicates TFEB as a functional downstream mediator of ANGPTL4 [66].

TFEB impairment was accompanied by functional lysosomal defects. ANGPTL4 overexpression increased Lysosomal pH and reduced the activities of both cathepsin B and LAL. An acidic lysosomal lumen is essential for optimal hydrolase activity, and recent studies have shown that lysosomal alkalinization compromises cathepsin B activity, including direct evidence obtained using the Magic Red assay, whereas restoration of lysosomal acidification improves lysosomal degradative and lipophagic function [44, 67]. During lipophagy, LAL catalyzes the intralysosomal hydrolysis of triglycerides and cholesteryl esters derived from lipid droplets, thereby facilitating terminal lipid degradation [68, 69]. Although reduced lipid droplet–autophagosome colocalization suggests that earlier stages of lipophagy may also be affected, the increased lysosomal pH, reduced LAL activity, and ultrastructural evidence of retained lipid droplets identify defective intralysosomal degradation as a major site of blockade. These findings provide a mechanistic explanation for the accumulation of BODIPY‐positive lipid material within enlarged lysosomal compartments. Nevertheless, the signaling events linking ANGPTL4 to TFEB cytoplasmic retention remain to be determined.

Moreover, HGPA stimulation increased both intracellular ANGPTL4 expression and the release of its full‐length (45 kDa) and cleaved (35 kDa) forms in a concentration‐dependent manner. Given the potentially distinct biological activities of these forms, future studies should define their respective extracellular and intracellular functions in podocytes. A long‐standing clinical observation is that renal lipid deposition in DKD can occur independently of systemic hyperlipidemia, pointing to local intrarenal regulatory mechanisms [19, 70]. Our findings now provide a mechanistic basis for this phenomenon. Seminal work by Clement et al. established that the proteinuric effect of ANGPTL4 depends mainly on its local renal expression, particularly in podocytes, rather than its circulating levels [26, 27, 31, 71, 72, 73, 74]. We extend this concept by showing that HGPA exposure not only increases intracellular ANGPTL4 but also promotes the release of full‐length and cleaved ANGPTL4 from podocytes. Recombinant ANGPTL4 recapitulated the reduction in nuclear TFEB, defective lysosomal acidification, and impaired lipid clearance, whereas extracellular neutralization partially reversed these changes. These findings support a functional contribution of secreted ANGPTL4 to lysosomal dysfunction in podocytes under diabetic conditions. The incomplete rescue achieved by neutralization, however, leaves open the possibility that intracellular ANGPTL4 also contributes, while the relative roles of full‐length and cleaved ANGPTL4 remain to be defined. This local lysosomal mechanism is distinct from the systemic hypertriglyceridemia caused by circulating ANGPTL4‐mediated inhibition of lipoprotein lipase, underscoring the importance of tissue‐specific context [75].

Together with reported roles in diabetic vascular injury, our findings highlight the context‐dependent actions of ANGPTL4 across diabetic complications. In podocytes, ANGPTL4 impairs autophagic–lysosomal clearance, whereas in vascular cells it has been linked to endothelial injury and fibrotic responses [76]. These observations raise the possibility that appropriately targeted modulation of ANGPTL4 may have relevance across diabetic microvascular and macrovascular disease.

Beyond impaired lipid clearance, suppression of the TFEB–lysosome axis was accompanied by podocyte dedifferentiation and profibrotic remodeling. In cultured podocytes, ANGPTL4 overexpression increased SQSTM1/p62 accumulation, reduced the LC3B‐II/LC3B‐I ratio and synaptopodin expression, and enhanced COL3A1 abundance. TFEB‐S211A attenuated the accumulation of collagen I, α‐smooth muscle actin, fibronectin, and collagen III under HGPA conditions, supporting a functional link between lysosomal dysfunction and the profibrotic phenotype. Consistently, glomeruli with high ANGPTL4 expression in human DKD biopsies exhibited greater accumulation of these extracellular matrix and mesenchymal‐associated markers. These findings suggest that persistent impairment of lysosomal clearance contributes to podocyte maladaptation and renal fibrotic progression.

Several limitations should be acknowledged. First, the mechanistic experiments were primarily performed in immortalized human podocytes, and the ANGPTL4–TFEB–lysosome axis remains to be validated in podocyte‐specific in vivo models. Second, the limited cohort size and the potential confounding effect of concurrent tubulointerstitial nephritis also preclude conclusions regarding the independent prognostic value of renal ANGPTL4 expression. Larger prospective cohorts with appropriate adjustment for baseline renal function, proteinuria, pathological severity, tubulointerstitial inflammation, and treatment‐related factors will be required to determine whether renal ANGPTL4 independently predicts DKD progression.

Our study identifies restoration of lysosomal function and lipophagic flux as potential therapeutic objectives in DKD. Strategies directed toward renal ANGPTL4 inhibition or TFEB activation may enhance lysosomal capacity, reduce podocyte lipotoxicity, and attenuate profibrotic remodeling. Future studies using podocyte‐specific in vivo models and isoform‐selective interventions will be essential to establish the therapeutic feasibility and safety of targeting this pathway. Collectively, our findings position the ANGPTL4–TFEB–lysosome axis as a mechanistic link between diabetic metabolic stress, defective lipid clearance, and progressive podocyte injury.

## 4 Conclusion

In summary, our data support a model in which renal ANGPTL4 expression under diabetic metabolic stress is associated with impaired TFEB‐mediated lysosomal function and lipophagic clearance, leading to lipid accumulation, podocyte injury, and profibrotic remodeling in vitro. The clinical cohort demonstrates associations between renal ANGPTL4 expression and DKD severity indices, whereas additional podocyte‐specific in vivo studies will be required to establish causal renal effects and therapeutic potential.

## 5 Experimental Section

### 5.1 Patients and Renal Tissue Samples

This retrospective study included patients pathologically diagnosed with DKD at China–Japan Friendship Hospital between January 2010 and December 2024. Inclusion criteria were: (1) age 18–75 years; (2) clinical diagnosis of type 1 or type 2 diabetes; (3) renal biopsy confirming DKD; and (4) a minimum follow‐up duration of one year. Patients with coexisting non‐DKD renal pathologies or incomplete data were excluded.

Follow‐up extended from the biopsy date until the occurrence of end‐stage renal disease (ESRD, defined as eGFR <15 mL/min/1.73 m^2^ or initiation of renal replacement therapy), death, loss to follow‐up, or the study cutoff date (December 2024). Normal control renal tissues (n = 5) were obtained from regions distant to tumors in nephrectomy specimens, confirmed to be histologically normal. The study protocol was approved by the Ethics Committee of China‐Japan Friendship Hospital (Approval No. 2024‐KY‐360).

### 5.2 Histology, Immunohistochemistry, and Immunofluorescence

Formalin‐fixed, paraffin‐embedded renal sections (3 µm) were deparaffinized and rehydrated. For immunohistochemistry (IHC), antigen retrieval was performed in Tris‑EDTA buffer (pH 9.0) using a microwave. Endogenous peroxidase activity was quenched with 3% H_2_O_2_. Sections were incubated with primary antibodies, followed by HRP‐conjugated secondary antibodies (PV‐9000; Zhongshan Golden Bridge Biotechnology, Beijing, China) and developed with 3,3′‐diaminobenzidine (DAB). For immunofluorescence (IF), sections were blocked with 10% goat serum and incubated with primary antibodies overnight at 4°C, then with appropriate fluorophore‐conjugated secondary antibodies for 1 h at room temperature. Masson's trichrome staining was performed according to the manufacturer's instructions (KTMTR; StatLab, McKinney, TX, USA). Brightfield slides were scanned using a G Cell Gscan‑12 whole‐slide scanner. Confocal images were acquired using a STELLARIS 5 microscope (Leica Microsystems, Wetzlar, Germany).

### 5.3 Reagents and Antibodies

The following reagents were used: high‐fat diet (D12492; Research Diets, New Brunswick, NJ, USA), streptozotocin (S0130; Sigma‐‑Aldrich, St. Louis, MO, USA), adriamycin (D1515; Sigma–Aldrich), AGE‐BSA (ab51995; Abcam, Cambridge, MA, USA), palmitic acid (P0500; Sigma‑‐Aldrich), chloroquine diphosphate (C6628; Sigma–Aldrich), bafilomycin A1 (B1793; Sigma Aldrich), rapamycin (R8781; Sigma–Aldrich), Lipofectamine 3000 (L3000015; Thermo Fisher Scientific), recombinant human ANGPTL4 N‐terminal fragment (8249‐AN; R&D Systems), monensin (S1753; Beyotime), nigericin (Y261764; Beyotime), Anti‐ANGPTL4 (A2772; Selleck Chemicals) and NE‐PER Nuclear and Cytoplasmic Extraction Reagents (78833; Thermo Fisher Scientific).

Antibodies for immunoblotting and staining included: anti‐ANGPTL4 (51109‐1‐AP, dilution 1:1000; Proteintech, Rosemont, IL, USA), anti‐SQSTM1/p62 (ab56416, dilution 1:2000; Abcam), anti‐LC3B (ab232940, dilution 1:1000; Abcam), anti‐LAMP1 (sc‐20011, dilution 1:200; Santa Cruz Biotechnology, Dallas, TX, USA), anti‐nephrin (GP‐N2, dilution 1:100; Progen, Heidelberg, Germany), anti‐synaptopodin (67339‐1‐Ig, dilution 1:2000; Proteintech), anti‐α‐SMA (67735‐1‐Ig, dilution 1:1000; Proteintech), anti‐fibronectin (ab2413, dilution 1:500; Abcam), anti‐collagen I (ab138492, dilution 1:1000; Abcam), anti‐collagen III (ab184993, dilution 1:1000; Abcam), anti‐β‐actin (A5316, dilution 1:10000; Sigma–Aldrich), anti‐Lamin B1 (ab133741; Abcam), anti‐Histone H3 (17168‐1‐AP; Proteintech), anti‐TFEB (ab270604; Abcam), anti‐phospho‐TFEB (Ser211) (ab310331; Abcam), and anti‐α‐tubulin (11224‐1‐AP, dilution 1:10000; Proteintech).

Secondary antibodies for immunofluorescence were from Jackson ImmunoResearch (West Grove, PA, USA). DAPI (ab104139, 1 µg/mL; Abcam), LysoTracker Deep Red (L12492, 50 nM; Thermo Fisher Scientific, Waltham, MA, USA), Bodipy 493/503 (D3922, 1 µM; Thermo Fisher), LysoSensor Yellow/Blue DND‐160 (L7545; Thermo Fisher Scientific), Cathepsin B Assay Kit (Magic Red; ab270772; Abcam), LysoLive Lysosomal Acid Lipase Assay Kit (ab253380; Abcam), and the RFP‐GFP‐LC3B tandem fluorescent protein kit (P36239; Thermo Fisher) were used for live‐cell imaging.

Normal renal tissue samples were obtained from five age‐ and sex‐matched patients who underwent nephrectomy for renal tumors. These control tissues were taken from regions distant from the tumor site and confirmed by pathological examination to be free of renal abnormalities.

The study protocol was reviewed and approved by the Ethics Committee of China‐Japan Friendship Hospital in compliance with ethical standards for retrospective research (Approval No. 2024‐KY‐360).

### 5.4 Image Analysis

Image quantification was performed using Fiji/ImageJ software (National Institutes of Health, Bethesda, MD, USA). ANGPTL4 expression was quantified as the mean fluorescence intensity within the glomerular and tubulointerstitial compartments, analyzing at least five glomeruli per section. LC3B intensity and SQSTM1/p62 accumulation in podocytes were measured similarly. Colocalization analysis (e.g., LC3B with SQSTM1) was conducted by calculating Pearson's correlation coefficient using the built‐in coloc2 plugin. To stratify patients based on glomerular ANGPTL4 expression, fluorescence intensity was measured in five random glomeruli per section. A receiver operating characteristic (ROC) curve determined the optimal cutoff value to define ANGPTL4‐high and ANGPTL4‐low patient subgroups.

### 5.5 Transcriptomic and Bioinformatic Analysis

Two public Gene Expression Omnibus (GEO) datasets were analyzed: bulk RNA‐seq dataset GSE30528 (9 DKD vs. 13 normal controls) and single‐nucleus RNA‐seq dataset GSE211785 (3 DKD, 8 HKD, and 7 control samples). Data processing included quality control, normalization, and dimensionality reduction (UMAP, PCA). Cell types were identified using established marker genes. GSEA was performed against autophagy and lipid metabolism gene sets from the Molecular Signatures Database (MSigDB). All analyses were conducted in R (version 4.3.1) and GraphPad Prism (version 9.0), with statistical significance set at *p* < 0.05.

### 5.6 Animal Models

All animal experiments were approved by the Experimental Animal Ethics Committee of China–Japan Friendship Hospital (Approval No. ZRDWLL240045). For the diabetic rat model, male Sprague‐Dawley rats (200–250 g) were fed a high‐fat diet (HFD; 60% kcal from fat) for two weeks, followed by a single intraperitoneal injection of STZ (35 mg/kg in citrate buffer, pH 4.5). Rats with sustained non‐fasting blood glucose ≥16.7 mmol/L were considered diabetic and maintained on HFD for up to 4 months post‐induction.

For the genetic diabetic mouse model, male *db/db* mice and their heterozygous non‐diabetic littermates (*db/m* mice) were purchased from Cyagen Biosciences (Suzhou, China). Mice were maintained under specific pathogen‐free conditions with free access to standard chow and water. Kidney tissues were harvested at two time points representing early and advanced stages of diabetes: 16 weeks and 28 weeks of age.

For the adriamycin‐induced mouse model, male BALB/cJ mice (7 weeks old) received a single tail‐vein injection of adriamycin (ADR, 10 mg/kg). Control mice received an equivalent volume of normal saline. Kidney tissues were harvested one week post‐injection for analysis.

### 5.7 Transmission Electron Microscopy (TEM)

Renal biopsy specimens or pelleted cultured podocytes were fixed in 2.5% glutaraldehyde in 0.1 M phosphate buffer, post‐fixed in 1% osmium tetroxide, and embedded in Epon 812 resin. Ultrathin sections (70 nm) were stained with uranyl acetate and lead citrate. Images were acquired using a JEM‐1400 Flash transmission electron microscope (JEOL Ltd., Tokyo, Japan).

### 5.8 Cell Culture and Treatments

Conditionally immortalized human podocytes were cultured under permissive conditions as previously described. To model diabetic lipotoxicity, cells were treated with HGPA (final concentrations: 30 mM D‐glucose and 300 µM sodium palmitate complexed with fatty acid‐free BSA at a 5:1 molar ratio) for 24 h. Control groups included: normal glucose medium (5.5 mM D‐glucose), isotonic osmotic control (5.5 mM glucose + 24.5 mM mannitol), high glucose alone (30 mM, 72 h), and AGE‐BSA (200 µg/mL, 24 h).

### 5.9 Autophagic Flux Assay

Autophagic flux was monitored using the RFP‐GFP‐LC3B tandem fluorescent reporter system. Podocytes were transfected with the reporter construct and subsequently treated with CQ (25 µM, 4 h), Baf A1(100 nM, 4 h), or rapamycin (100 nM, 6 h) as indicated. Cells were imaged live, and the numbers of GFP^+^RFP^+^ puncta (autophagosomes) and GFP^−^RFP^+^ puncta (autolysosomes) per cell were quantified.

### 5.10 Plasmid Transfection and Viral Transduction

For transient overexpression, podocytes were transfected with ANGPTL4 expression plasmid or corresponding empty vector using Lipofectamine 3000 (L3000015; Thermo Fisher). For stable knockdown, cells were transduced with lentiviral particles encoding ANGPTL4‐targeting shRNA (sequences: #1: 5′‐GAAGCTTAAGAAGGGAATCTT‐3′; #2: 5′‐GACCACAAGCACCTAGACCAT‐3′; #3: 5′‐GAACAGCAGGATCCAGCAACT‐3′) in the presence of polybrene (8 µg/mL; TR‐1003; Sigma) and selected with puromycin (2 µg/mL). Adenoviruses expressing wild‐type TFEB or the constitutively active mutant TFEB‐S211A were used at a multiplicity of infection (MOI) of 50.

### 5.11 Western Blot and Secretome Analysis

Cells were lysed in SDS buffer (62.5 mM Tris‐HCl, pH 6.8, 2% SDS, 10% glycerol) supplemented with protease inhibitors. Protein concentration was determined by BCA assay. Equal amounts of protein (10 µg) were separated by SDS‐PAGE, transferred to PVDF membranes, blocked, and incubated with primary and HRP‐conjugated secondary antibodies. Signals were developed using enhanced chemiluminescence substrate. For secreted ANGPTL4 analysis, conditioned medium was concentrated using Amicon Ultra centrifugal filters (10 kDa cutoff; UFC9010; Millipore, Burlington, MA, USA). Total protein in the concentrate was assessed by Ponceau S staining for normalization.

### 5.12 Live‐Cell Imaging and Lipophagy Quantification

Podocytes were transfected with an LC3B‐RFP plasmid to label autophagosomes. Neutral lipid droplets were stained with Bodipy 493/503. Where indicated, cells were pre‐treated with rapamycin (100 nM, 6 h) and/or chloroquine (50 µM, 4 h). Live‐cell confocal imaging was performed at 37°C under 5% CO_2_. The number, average size, and total area of lipid droplets per cell were quantified using the “Analyze Particles” function in ImageJ. Colocalization between LC3B‐RFP and Bodipy signals was assessed by calculating Pearson's correlation coefficient.

### 5.13 Immunofluorescence in Cultured Podocytes

Podocytes grown on glass coverslips were fixed with 4% paraformaldehyde, permeabilized with 0.3% Triton X‐100, and blocked with 10% fetal bovine serum. Cells were incubated with primary antibodies overnight at 4°C, followed by fluorophore‐conjugated secondary antibodies and DAPI. Images were captured using a confocal microscope.

#### 5.13.1 RNA Isolation and Quantitative Real‐Time PCR (qPCR)

Total RNA was extracted using TRIzol reagent. cDNA was synthesized using a reverse transcription kit, and qPCR was performed with SYBR Green master mix on a QuantStudio5 Real‐Time PCR System. GAPDH served as the internal reference gene. Primer sequences were: GAPDH‐F: CTCTGCTCCTCCTGTTCGAC, GAPDH‐R: GCGCCCAATACGACCAAATC; ANGPTL4‐F: CCACCGACCTCCCGTTAG, ANGPTL4‐R: TTCTGAGCCTTGAGTTGTGTCT. Relative expression was calculated using the 2^(‐ΔΔCt) method.

#### 5.13.2 RNA Sequencing and Differential Expression Analysis

Total RNA from podocytes was assessed for purity and integrity. Libraries were prepared and sequenced on an Illumina NovaSeq platform 6000. After quality control, clean reads were aligned to the human reference genome (GRCh38). Gene expression was quantified, and differential expression analysis was performed using DESeq2. Genes with an absolute log2 fold change >1 and an adjusted *p*‐value < 0.05 were considered significantly differentially expressed.

### 5.14 Nuclear‐Cytoplasmic Fractionation and TFEB Immunoblotting

Podocytes were washed twice with ice‐cold PBS and harvested by gentle scraping. Nuclear and cytoplasmic proteins were isolated using the NE‐PER Nuclear and Cytoplasmic Extraction Reagents according to the manufacturer's protocol. Briefly, a packed cell pellet was sequentially extracted with Cytoplasmic Extraction Reagents I and II, followed by centrifugation to collect the cytoplasmic supernatant. The remaining nuclear pellet was resuspended in Nuclear Extraction Reagent, vortexed intermittently for 40 min on ice, and centrifuged at 16 000 × *g* for 10 min at 4°C to obtain the nuclear fraction. Protease and phosphatase inhibitors were included in all extraction buffers. Equal amounts of nuclear and cytoplasmic proteins were separated by SDS‐PAGE and immunoblotted for TFEB. Lamin B1 or Histone H3 served as nuclear loading controls, whereas alpha‐tubulin served as the cytoplasmic loading control. Band intensities were quantified using Fiji/ImageJ and normalized to the corresponding compartment‐specific loading control.

### 5.15 TFEB Immunofluorescence and Nuclear Localization Analysis

Podocytes grown on glass‐bottom dishes were fixed with 4% paraformaldehyde for 15 min, permeabilized with 0.3% Triton X‐100 for 10 min, and blocked with 5% bovine serum albumin for 1 h at room temperature. Cells were incubated with anti‐TFEB antibody (ab270604; 1:200) overnight at 4°C, followed by an appropriate fluorophore‐conjugated secondary antibody for 1 h at room temperature. Nuclei were counterstained with DAPI, and images were acquired using a Leica STELLARIS 5 confocal microscope under identical acquisition settings. Nuclear masks were generated from the DAPI channel, and whole‐cell boundaries were manually delineated using the TFEB and transmitted‐light images. Nuclear TFEB was calculated as the background‐subtracted integrated TFEB intensity within the nuclear mask divided by the background‐subtracted integrated TFEB intensity of the whole cell and expressed as a percentage. Cells were pooled from three independent experiments, and each point represents one analyzed cell.

### 5.16 Extracellular ANGPTL4 Stimulation, Neutralization, and TFEB Rescue

Podocytes were treated with recombinant human N‐terminal ANGPTL4 fragment at a final concentration of 1 µg mL^−1^ for 24 h. This condition was selected based on previously reported biologically active concentrations and preliminary optimization experiments [77, 78]. Vehicle‐treated cells received an equal volume of sterile PBS. For extracellular ANGPTL4 blockade, podocytes were pretreated with an anti‐ANGPTL4 monoclonal antibody (10 µg mL^−1^; A2772, Selleck Chemicals) for 24 h. recombinant human N‐terminal ANGPTL4 fragment was then added at a final concentration of 1 µg mL^−1^, and the cells were incubated for an additional 24 h in the continued presence of the antibody before subsequent analyses. For TFEB rescue, podocytes were transduced with an adenovirus encoding the constitutively active TFEB‐S211A mutant at a multiplicity of infection of 50. After 24 h of transduction, recombinant Human N‐terminal ANGPTL4 fragment was added at a final concentration of 1 µg mL^−1^, and the cells were cultured for an additional 24 h before subsequent analyses.

### 5.17 Determination of Lysosomal pH

For confocal fluorescence imaging of LysoSensor Yellow/Blue DND‐160 in podocytes across different treatment groups, after completing transfection and drug treatment, the probe was diluted to 2 µm in DMEM. Cells were incubated in the pre‐warmed probe solution at 37°C for 1 h, followed by PBS washing. Pre‐warmed DMEM was then added to the cells before image acquisition using a confocal microscope (STELLARIS 5, Leica Microsystems, Wetzlar, Germany). Fluorescence images were captured at emission wavelengths of 450 ± 33 nm (blue fluorescence, representing less acidic lysosomal environments) and 510 ± 20 nm (yellow fluorescence, indicating more acidic lysosomal compartments) [44, 79]. Using Fiji software, the yellow and blue fluorescence dots were measured for each cell.

For absolute lysosomal pH measurement in podocytes, cells were seeded in 96‐well plates and incubated with 2 µM LysoSensor Yellow/Blue DND‐160 diluted in DMEM at 37°C for 3–5 min, followed by PBS washing. Cells were then briefly rinsed with PH calibration buffers (PH 4–7) containing 10 µM monensin (S1753, Beyotime, China) and 30 µM nigericin (Y261764, Beyotime, China) and immediately incubated with 100 µL of respective PH standard buffers at 37°C for 10 min. Fluorescence intensity was measured using a microplate reader (Tecan, Männedorf, Switzerland) with dual excitation/emission settings Ex  =  329 nm/Em  =  440 nm and E x  =  384 nm/Em  =  540 nm. A standard curve was generated by plotting the fluorescence intensity ratio ex: 329/380 against known PH values, enabling calculation of absolute Lysosomal pH in test samples based on their measured fluorescence ratios interpolated against this calibration curve.

### 5.18 Cathepsin B Activity Assay

Cathepsin B activity was assessed in live podocytes using the Magic Red Cathepsin B Assay Kit (ab270772; Abcam), following the manufacturer's protocol and a previously reported lysosomal enzyme assay. Podocytes were incubated for 30 min at 37°C protected from light. Cells were then washed twice with PBS and imaged immediately using a Leica STELLARIS 5 confocal microscope. Magic Red fluorescence was excited at 561 nm and collected in the red emission range. Mean Magic Red fluorescence intensity was measured within the whole‐cell region after background subtraction. Ten cells per group, pooled from three independent experiments, were analyzed.

### 5.19 Lysosomal Acid Lipase Activity by LipaGreen Imaging and Flow Cytometry

Adherent podocytes were incubated with the staining medium for 6 h at 37°C. For confocal analysis, cells were washed with PBS and imaged live using a Leica STELLARIS 5 confocal microscope with 488‐nm excitation and emission collection at approximately 500–550 nm. Mean LipaGreen fluorescence intensity was quantified after background subtraction in ten cells per group pooled from three independent experiments. For flow cytometry, stained cells were detached using 0.25% trypsin‐EDTA, collected by centrifugation at 350 × *g* for 5 min, and resuspended in 1× Flow Holding and Sorting Buffer for adherent cells. Samples were analyzed using a 488‐nm laser and the FL1/FITC detector at approximately 520 nm. Debris, doublets, and nonviable cells were excluded using standard forward‐scatter, side‐scatter, singlet, and viability gates, and at least 10 000 live singlet events were acquired per sample. LAL activity was represented by the median fluorescence intensity of LipaGreen and normalized to the vector‐control group. The procedure was based on the manufacturer's protocol and a recent Nature Communications study using LipaGreen to evaluate lysosomal lipid degradation.

### 5.20 BODIPY‐LysoTracker Co‐staining and Colocalization Analysis

To evaluate the lysosomal handling of lipid droplets, live podocytes were incubated with BODIPY 493/503 (1 micromolar) and LysoTracker Deep Red (50 nanomolar) for 30 min at 37°C. After washing, cells were imaged immediately in pre‐warmed phenol‐red‐free medium using a Leica STELLARIS 5 confocal microscope under identical acquisition settings. Lipid droplets were segmented from the BODIPY channel using a fixed background‐subtracted threshold, and total lipid‐droplet area per cell was calculated using the Analyze Particles function in Fiji/ImageJ. LysoTracker‐positive structures were segmented using the same thresholding strategy and mean lysosomal area per cell was calculated. Colocalization was assessed using the Coloc2 plugin. Manders’ M1 coefficient was defined as the fraction of BODIPY‐positive fluorescence intensity overlapping with LysoTracker‐positive signal. Each point represents one cell, and cells were pooled from three independent experiments.

### 5.21 Statistical Analysis

Statistical analyses were performed using SPSS software (version 20.0; IBM Corp., Armonk, NY, USA) and GraphPad Prism software (version 9.0; GraphPad Software, San Diego, CA, USA). Data are presented as the mean ± standard deviation (SD) for normally distributed continuous variables or as the median with interquartile range (IQR) for non‐normally distributed variables. Unless otherwise indicated, experiments were performed independently at least three times, and the exact sample size (n) is specified in the corresponding figure legends. Data distribution was assessed using the Shapiro–Wilk test. Comparisons between two independent groups were performed using an unpaired two‐tailed Student's t‐test for normally distributed data or the Mann–Whitney U test for non‐normally distributed data. Comparisons among multiple groups were performed using one‐way analysis of variance (ANOVA) followed by Tukey's post hoc test. Pearson's correlation analysis was used for normally distributed variables with a linear relationship, whereas Spearman's rank correlation analysis was used for non‐normally distributed variables. All statistical tests were two‐sided, and *p* < 0.05 was considered statistically significant. Statistical significance is indicated as follows: ns, not significant; *p < 0.05, **p < 0.01, ***p < 0.001, ****p < 0.0001.

## Author Contributions

X.L. and S.J. conceived the study and designed the experiments. X.L., S.S., and J.L. collected and analyzed the data. Z.Y. conducted the bioinformatic and statistical analysis. S.J. and C.Z. performed animal models. X.L., J.L and G.Z. performed in vitro experiments. S.J. and W.L. supervised the entire research project, acquired funding, and oversaw the manuscript preparation. X.L. wrote the original draft. S.J. approved the final version of the manuscript. All authors read and approved the final paper.

## Funding

This research was funded by the National High Level Hospital Clinical Research Funding (2025‐NHLHCRF‐JBGS‐A‐WZ‐05 to W.L.), the National Natural Science Foundation of China (82300815 to S.J.), and the China Health Promotion Foundation (DKD‐MBD project, 2018‐HX‐86 to W.L.).

## Conflicts of Interest

The authors declare no conflicts of interest.

## Supporting information




**Supporting File**: advs76771‐sup‐0001‐SuppMat.docx.

## Data Availability

The data that support the findings of this study are available from the corresponding author upon reasonable request.
