# Supplementary material for: ANGPTL4 Exacerbates Renal Injury in Diabetic Kidney Disease by Impairing Podocyte Lipophagy via Compromised Lysosomal Degradative Function
Source: Adv Sci (Weinh). 2026 Jul 27;13(58):e76771. doi: 10.1002/advs.76771 (PMC13403719; doi:10.1002/advs.76771)
Supplement: Supplementary file 1 [file ADVS-13-e76771-s001.docx]

**Supporting Information**

**ANGPTL4 Exacerbates Renal Injury in Diabetic Kidney Disease by Impairing Podocyte Lipophagy via Compromised Lysosomal Degradative Function**

*Xiaojing Liu^1^, Shimin Jiang^1^*, Shunlai Shang^1^, Zhenkun Yang^2^, Jiayi Li^3^, Guming Zou^1^, Cheng Zhou^1^, and Wenge Li^1^**

Affiliations

^1^ Department of Nephrology, China-Japan Friendship Hospital, Beijing, 100029, China

^2^ China-Japan Friendship Hospital (institute of Clinical Medical Sciences), Chinese academy of Medical Sciences & Peking union Medical College, Beijing 100029, China

^3^ Health Science Center, Peking University, Beijing, 100191, China

*Corresponding author

jiangshimin@zryhyy.com.cn (S. Jiang)

liwenge@pumc.edu.cn (W. Li)

**Figure S1. Flowchart of the study population**

**Figure S2. Podocyte-specific suppression of autophagy pathways in DKD revealed by snRNA-seq.** **Related to Figure 1.**

(A) Uniform Manifold Approximation and Projection (UMAP) of snRNA-seq data (GSE211785) from human kidney samples, including diabetic kidney disease (DKD, n=3), healthy controls (n=7), and hypertensive kidney disease (HKD, n=8).

(B) Top 15 enriched pathways from Gene Ontology (GO, left) and Kyoto Encyclopedia of Genes and Genomes (KEGG, right) analyses across all cells. The autophagy-related pathway is marked with an asterisk.

(C) Cell-specific analysis shows that autophagy-related GO pathways are significantly downregulated in podocytes, but not in tubular cells, of DKD patients compared to both control groups.

**Figure S3. Patient stratification based on glomerular ANGPTL4 immunofluorescence.** **Related to Figure 2 and Figure 6.**

The optimal cutoff for classification was determined by analyzing glomerular ANGPTL4 immunofluorescence intensity using receiver operating characteristic (ROC) curve analysis.

**Figure S4. Podocyte lipid droplets are absent in STZ/HFD-induced and genetic diabetic models but present in an ADR-induced nephropathy model, related to Figure 3.**

Representative transmission electron microscopy (TEM) images showing model-specific patterns of intracellular lipid deposition. (A) In streptozotocin/high-fat diet (STZ/HFD)-induced diabetic rats, lipid droplets (LDs, white arrows) accumulated exclusively in tubular epithelial cells at the indicated time points (0, 2, and 4 months post-model induction), with no detectable LDs in podocytes (n=3 per group). In contrast, prominent podocyte LDs were evident in adriamycin (ADR)-treated mice as early as one week post-injection. (B) In genetic diabetic db/db mice, no podocyte LDs were observed at either 16 or 28 weeks of age, despite the presence of characteristic diabetic glomerulopathies, including glomerular basement membrane (GBM) thickening and foot process effacement (FPE). Their heterozygous db/m littermates served as controls.

Scale bars: 1 μm. Abbreviations: ADR, adriamycin; GBM, glomerular basement membrane; FPE, foot process effacement; LDs, lipid droplets; Mit, mitochondria; STZ/HFD, streptozotocin/high-fat diet; TEM, transmission electron microscopy.
